# PrP is a central player in toxicity mediated by soluble aggregates of neurodegeneration-causing proteins

**DOI:** 10.1007/s00401-019-02114-9

**Published:** 2019-12-18

**Authors:** Grant T. Corbett, Zemin Wang, Wei Hong, Marti Colom-Cadena, Jamie Rose, Meichen Liao, Adhana Asfaw, Tia C. Hall, Lai Ding, Alexandra DeSousa, Matthew P. Frosch, John Collinge, David A. Harris, Michael S. Perkinton, Tara L. Spires-Jones, Tracy L. Young-Pearse, Andrew Billinton, Dominic M. Walsh

**Affiliations:** 1 Laboratory for Neurodegenerative Research, Ann Romney Center for Neurologic Diseases, Brigham and Women’s Hospital and Harvard Medical School, Hale Building for Transformative Medicine, 60 Fenwood Road, Boston, MA 02115 USA; 2 Centre for Discovery Brain Sciences and UK Dementia Research Institute, University of Edinburgh, Edinburgh, EH89JZ UK; 3 Program for Interdisciplinary Neuroscience, Brigham and Women’s Hospital, Boston, MA 02115 USA; 4 Massachusetts General Institute for Neurodegenerative Disease, Massachusetts General Hospital and Harvard Medical School, Charlestown, MA 02129 USA; 5 MRC Prion Unit at UCL, UCL Institute of Prion Diseases, National Hospital for Neurology and Neurosurgery, Queen Square, London, WC1N 3BG UK; 6 Department of Biochemistry, Boston University School of Medicine, Boston, MA 02118 USA; 7 Neuroscience, IMED Biotechnology Unit, AstraZeneca, Cambridge, CB21 6GH UK

**Keywords:** Aβ, Alzheimer’s disease, α-Synuclein, Dementia with Lewy bodies, Prion protein, Tau

## Abstract

Neurodegenerative diseases are an enormous public health problem, affecting tens of millions of people worldwide. Nearly all of these diseases are characterized by oligomerization and fibrillization of neuronal proteins, and there is great interest in therapeutic targeting of these aggregates. Here, we show that soluble aggregates of α-synuclein and tau bind to plate-immobilized PrP in vitro and on mouse cortical neurons, and that this binding requires at least one of the same N-terminal sites at which soluble Aβ aggregates bind. Moreover, soluble aggregates of tau, α-synuclein and Aβ cause both functional (impairment of LTP) and structural (neuritic dystrophy) compromise and these deficits are absent when PrP is ablated, knocked-down, or when neurons are pre-treated with anti-PrP blocking antibodies. Using an all-human experimental paradigm involving: (1) isogenic iPSC-derived neurons expressing or lacking *PRNP*, and (2) aqueous extracts from brains of individuals who died with Alzheimer’s disease, dementia with Lewy bodies, and Pick’s disease, we demonstrate that Aβ, α-synuclein and tau are toxic to neurons in a manner that requires PrP^C^. These results indicate that PrP is likely to play an important role in a variety of late-life neurodegenerative diseases and that therapeutic targeting of PrP, rather than individual disease proteins, may have more benefit for conditions which involve the aggregation of more than one protein.

**Electronic supplementary material:**

The online version of this article (10.1007/s00401-019-02114-9) contains supplementary material, which is available to authorized users.

## Introduction

The presence of macroscopic protein aggregates is pathognomonic for most common neurodegenerative disorders [78, 82]. Alzheimer’s disease (AD) is characterized by two types of protein deposits: plaques composed of amyloid β-protein (Aβ) and tau-containing neurofibrillary tangles [36, 59]. Tau aggregates are also found in progressive supranuclear palsy (PSP), corticobasal degeneration (CBD) and Pick’s disease (PiD) [5, 50, 70]. In Parkinson’s disease (PD) and dementia with Lewy bodies (DLB), α-synuclein (αSyn) accumulates within neurons as Lewy bodies and Lewy neurites [83]. The end-stage aggregates present in these various diseases are β-sheet-rich and fibrillar in nature, but have distinct ultrastructures [27, 28, 30, 57, 72]. Whether these thermodynamically stable aggregates drive pathogenesis or merely reflect aberrant processing of the parent proteins is controversial, and significant evidence suggests that soluble aggregates (often referred to as oligomers) are the primary mediators of neuronal dysfunction [18, 88].

Certain assemblies of Aβ, αSyn and tau have been shown to be potent neurotoxins [22, 24, 26, 29, 61, 67, 85], but to date the activity of Aβ assemblies has been the most intensively studied, with more than a dozen putative receptors proposed [8]. Of these candidates, the cellular prion protein (PrP^C^) has more supportive evidence than most [81]. All published studies on the Aβ-PrP interaction have concluded that binding is high affinity (sub-nanomolar) and oligomer-specific, and many have found that binding to PrP mediates a toxic response [19, 31, 32, 53, 62, 66, 73, 84, 91, 93]. However, there have been reports of deleterious effects of Aβ that do not require PrP^C^ expression [4, 15, 20, 51]. The likely explanation for these conflicting results lies in the lack of standardization of the Aβ preparations used and the unrealistic expectation that all forms of Aβ should cause toxicity by a PrP-dependent mechanism. Most studies that have investigated the Aβ–PrP interaction used Aβ-derived diffusible ligands (ADDLs) [19, 23, 31, 32, 66, 73, 84, 91, 93], a recipe-based preparation which contains monomers, protofibrils and globular oligomers [39, 52, 66]. However, we have shown that the duration of incubation used to generate ADDLs has dramatic effects on both binding to PrP and neurotoxicity [66]. Specifically, we found that fibrils and globular oligomers bind only weakly to PrP and mediate toxicity in a PrP^C^-independent manner. For instance, globular oligomers cause membrane leakage independent of the presence of PrP, and Aβ fibrils block LTP in slices from both wild-type (WT) and PrP-deficient mice. In contrast, soluble aggregates composed largely of protofibril-like structures block LTP in a PrP-dependent fashion [66].

Given the pleomorphic nature of Aβ assemblies formed in vitro [89] and those in extracts of human brain [25, 41, 92], it is reasonable to expect that toxicity is mediated by more than one Aβ assembly and that this could involve interactions between specific Aβ assemblies and specific receptors, such as PrP^C^ and rather non-specific membrane perturbations [87]. PrP^C^ is a membrane-associated protein best known for its role in transmissible spongiform encephalopathies [71]. Since the infectious component of these diseases is proteinaceous, they are also referred to as prion diseases. Prions reproduce by converting helical-rich PrP^C^ into β-sheet-rich infectious PrP^Sc^ [69]. Although initially unanticipated, the finding that PrP^C^ can serve as a receptor for oligomeric forms of Aβ [53] fits well with the known ability of PrP^C^ to bind misfolded PrP [6, 16] and opens up the tantalizing possibility that PrP may recognize a conformation shared by other disease-associated protein aggregates.

Recently, several studies have imputed a role for PrP^C^ in binding to, and mediating, αSyn and tau toxicity [29, 42, 67]. For tau, this association has been indirect, relying on certain antibodies to block PrP^C^ [42, 67] and in the case of αSyn there have been conflicting results [29, 86]. No studies have directly compared the interaction of αSyn, tau and Aβ with PrP or explored the activity of human brain-derived αSyn and tau. Here, we set out to systematically explore whether certain assemblies of tau and αSyn can bind to PrP and whether this binding has disease-relevant consequences. For this purpose, we developed a robust protocol that allows the reproducible generation of soluble protein aggregates (SPAs) of Aβ, tau and αSyn. Thereafter, we tested binding to PrP using a sensitive solid-phase ELISA-like assay, PrP deletion constructs, mouse primary neurons that express or lack PrP, iPSC-derived human neurons (iNs) with and without CRISPR-mediated deletion of *PRNP*, and aqueous extracts of AD, DLB and PiD brains. Importantly, SPAs of tau and αSyn bound to PrP with high affinity, but (as with Aβ) monomers and end-stage fibrils displayed little or no binding. Moreover, anti-PrP antibodies to the putative Aβ binding *Sites I* and *II* [19, 31, 32, 62] effectively displaced SPAs of αSyn and tau, and deletion of these sites substantially attenuated binding. Multielectrode recordings from hippocampal slices of PrP WT mice revealed that soluble aggregates (but not monomers) of Aβ, tau and αSyn impair LTP, and recordings from PrP null mice demonstrate that this impairment required expression of PrP. Additionally, high-content imaging and bioactivity assays utilizing primary mouse and iPSC-derived human neurons revealed that soluble aggregates of all three proteins interact with PrP on neuronal surfaces and exert dose- and time-dependent neuritotoxicity. In contrast, soluble aggregates of albumin bound only weakly to PrP and were not toxic to neurons. Most relevant to human disease, knock-out of PrP and pre-treatment with anti-PrP antibodies prevented toxicity of brain extracts from AD, PiD and DLB brains. Collectively, these results suggest that PrP plays an important role in brain proteinopathies and that targeting of PrP may offer a plausible means to treat such conditions.

## Materials and methods

### Chemicals, proteins and reagents

Human Aβ_1–42_ was synthesized and purified by Dr. James I. Elliott at the Yale University Keck Biotechnology Resource Laboratory (New Haven, CT). Peptide mass and purity (> 95%) was confirmed by electrospray ionization/ion trap mass spectrometry and reverse-phase HPLC. Full-length human α-synuclein (1–140) was kindly provided by Prof. Sara Linse (Lund University Center for Molecular Protein Science, Lund, Sweden) and murine PrP_91–231_ and PrP_119–231_ were graciously provided by Prof. John Collinge (University College London MRC Prion Unit, London, UK). The longest isoform of human tau (2N4R; hTau40) and murine PrP_23–231_ were purified in house and are detailed below. Aqueous paraformaldehyde was from Electron Microscopy Services (Hatfield, PA) and cell culture reagents were obtained from ThermoFisher (Carlsbad, CA). All other chemicals and reagents were of the highest purity available and were obtained from MilliporeSigma (St. Louis, MO) unless indicated otherwise.

### Antibodies

The antibodies, their sources and epitopes are described in Supplementary Table 2.

### Preparation of recombinant prion protein 23–231

*Escherichia coli* (*E. coli*) strain BL21(DE3)*p*LysS (New England BioLabs, Ipswich, MA) was transformed with the pTrcHis B vector expressing murine PrP_23–231_ [32, 44, 94]. Ampicillin-resistant colonies were selected, expanded and expression was induced by the addition of 1 mM IPTG for 16 h at 37 °C and 225 RPM. Cultures were harvested by centrifugation at 6000×*g* for 15 min, washed in PBS and lysed by sonication (2 × 120 s bursts at 30% output) in extraction buffer (50 mM Tris–HCl, pH 8.0, 200 mM NaCl, 0.1% Tween-20, 50 U/mL Benzonase, 10 μg/mL lysozyme). Suspensions were sedimented at 10,000×*g* for 30 min and the inclusion body-enriched pellets were extracted by sonication (2 × 120 s bursts at 30% output) in solubilization buffer (6 M GuHCl/50 mM Tris–HCl pH 8.0/0.8% β-mercaptoethanol). Suspensions were sedimented at 21,000×*g* for 45 min and the PrP-enriched supernatant was clarified with 5 µM and 0.45 µM syringe filters. Filtered supernatants were loaded onto 5 mL HisTrap HP columns (GE Life Sciences, Marlborough, MA) at 1 mL/min using a BioLogic DuoFlow FPLC system (Bio-Rad, Hercules, CA) and washed with 10 column volumes (CV) of Buffer A (6 M GuHCl, 10 mM Tris–HCl, 100 mM Na_2_HPO_4_, 10 mM Glutathione pH 8.0) at 1 mL/min. Bound PrP was refolded on-column in a linear gradient of Buffer A to Buffer B (10 mM Tris–HCl, 100 mM Na_2_HPO_4_, pH 8.0) for 30 CV at 0.213 mL/min (11 ¾ h). The following day, the column was eluted in a linear gradient of Buffer B to Buffer C (10 mM Tris–HCl, 100 mM Na_2_HPO_4_, 1 M imidazole, pH 5.8) for 3 CV at 0.5 mL/min and the fractions containing PrP were buffer exchanged with 2 kDa dialysis cassettes (ThermoFisher, Waltham, MA) overnight at against 1000 volumes of 20 mM Bis–Tris HCl, pH 6.5. The poly-histidine tag was cleaved from PrP using 50 U restriction-grade thrombin (Novagen, Madison, WI) overnight with agitation, and cleaved PrP was separated from the free histidine tag using a 5-mL HisTrap HP column. The fractions containing purified PrP were dialyzed overnight against 1000 volumes of 10 mM Bis–Tris HCl, pH 6.5. Protein purity was determined by SDS-PAGE/sliver staining and mass spectrometry, and secondary structure was analyzed by circular dichroism (Jasco J-815, Jasco, Easton, MD). Concentration was determined by measuring absorbance at 280 nm and using the predicted extinction coefficient (63,370, ε_280_ = M^−1^ cm^−1^).

### Circular dichroism

Secondary structure of PrP fragments was determined using a Jasco J-815 circular dichroism (CD) spectropolarimeter (Jasco Inc., Easton, MD). Proteins were buffer exchanged into 10 mM phosphate buffer, pH 7.4 using Zeba MicroSpin Desalting Columns (ThermoFisher, Waltham, MA) and diluted to 0.2 mg/mL for analysis. Near-UV signals were obtained by scanning from 260 to 180 nm at 50 nm/min with 2 s data integration time and 5 toggled accumulations. Each sample was measured in triplicate, and high tensor voltage never eclipsed 700 units across all measurements. Mean residue ellipticity was calculated from raw CD signals using the formula $\left[ {\uptheta } \right] = \frac{{100\left( {{\text{signal}}} \right)}}{{{\text{Cn}}l}}$, where [*θ*] = mean residue ellipticity in deg cm^2^ dmol^−1^; signal = raw output in mdeg; *C* = protein concentration in mM; *n* = number of residues; *l* = path length in cm [64].

### Preparation of recombinant Tau441 (hTau40)

Expression and purification of tau was performed essentially as described [47, 68]. *E. coli* strain BL21 (DE3) (New England BioLabs, Ipswich, MA) was transformed with a pNG2 vector encoding human tau 441 (2N4R) and protein expressed by the addition of 1 mM IPTG for 4 h at 37 °C and 225 RPM. After cell lysis, the soluble fraction containing tau was separated from insoluble material by boiling and ultracentrifugation, and tau was purified by anion exchange followed by two-step size exclusion chromatography. Purity of the proteins was assessed by SDS-PAGE/sliver staining and mass spectrometry. Concentration was determined by measuring absorbance at 280 nm and using the predicted extinction coefficient (ε_280_ = 7575 M^−1^ cm^−1^).

### Aggregation of Aβ, α-synuclein and tau

#### Aβ

One milligram lyophilisates were dispersed in denaturant 1 (7 M GuHCl and 5 mM EDTA), incubated overnight at room temperature, and monomer isolated by size exclusion chromatography (SEC) on a Superdex 75 10/300 column eluted with Aβ buffer (20 mM NaPO_4_, 0.2 mM EDTA, 0.02% NaN_3_, pH 8.0). Only the peak fractions were retained for aggregation assays. Aggregation was monitored using a continuous thioflavin-T (ThT) fluorescence assay performed essentially as described [38]. SEC-isolated peptide was diluted to 20.1 μM with Aβ buffer and combined with ThT to yield 30 μM ThT and 20 μM Aβ. Six replicates (100 μl) were transferred to wells of black, clear bottom, half-area PEG-ylated 96-well plates (#3881; Corning Life Sciences, Corning, NY). Reactions prepared in the absence of ThT were used for downstream fibril harvest, and blanks without any Aβ were included. Plates were covered with adhesive plate sealers (VWR, Radnor, PA) and incubated at 37 °C in a POLARstar Omega plate reader (BMG Labtech, Ortenberg, Germany). Fluorescence was recorded every 5 min (Ex_440_ and Em_480_) and reactions were carried out for at least 15 h.

#### α-Synuclein

Three-milligram lyophilisates were dispersed in denaturant 2 (6 M GuHCl, 25 mM Tris and 5 mM EDTA), incubated for 2 h at room temperature and chromatographed on a Superdex 75 10/300 column eluted with αSyn buffer (10 mM MES, 140 mM NaCl, pH 5.5). As with Aβ, only the peak fractions were retained for aggregation assays. Aggregation was monitored using a continuous ThT assay [35], with modification. SEC-isolated peptide was diluted to 40 μM with αSyn buffer and combined with ThT to yield 20 μM ThT and 35 μM αSyn. Six replicates (100 μL) were transferred to wells of black, clear bottom, full-area nontreated 96-well plates (#3631; Corning Life Sciences, Corning, NY). Reactions prepared in the absence of ThT were used for downstream fibril harvest, and blanks without any αSyn were included. Plates were covered with adhesive plate sealers (VWR, Radnor, PA) and incubated at 37 °C in a POLARstar Omega plate reader (BMG Labtech, Ortenberg, Germany) with shaking at 100 RPM for 300 s before each reading. Fluorescence was recorded every 10 min (Ex_440_ and Em_480_) and initial reactions were carried out for at least 96 h.

#### Tau

Monomeric tau was isolated by SEC using a Superdex 75 10/300 column eluted with tau buffer (138 mM NaCl, 2.7 mM KCl, 5 mM 1,4-dithiothreitol (DTT)) to reduce cysteine-mediated dimerization. As with Aβ and αSyn, only the peak fractions were retained for aggregation assays. Approximately 3–5 mg of tau in solution was concentrated to ≥ 60 µM using 10 kDa Amicon centrifugal filters (MilliporeSigma, St. Louis, MO) and buffer exchanged into 50 mM MES, pH 6.5 using 7 kDa Zeba desalting columns (ThermoFisher, Waltham, MA). Tau was diluted to 58.8 µM with 50 mM MES, pH 6.5, mixed with a 10X stock of DTT and heated at 55 °C for 10 min in protein lo-bind tubes (Eppendorf, Hamburg, Germany). Thereafter, the mixture was cooled to room temperature and heparin (#H4784; MilliporeSigma, St. Louis, MO) was added from a 20X stock so that the final aggregation reaction (in 250 µL) was: 50 µM tau, 100 µM DTT, 50 µM heparin. Tubes were incubated in a thermomixer (600 RPM) set to 37 °C and incubated for up to 12 days. Every third day, 10 µL aliquots from the aggregation reactions were removed and aggregation monitored by discontinuous ThT assay. Samples were diluted to 10 µM with MilliQ, supplemented with 10 µL 200 µM ThT and transferred to wells of black, clear bottom, half-area PEG-ylated 96-well plates (#3881; Corning Life Sciences, Corning, NY). Plates were incubated for 45 min with shaking at 300 RPM, and ThT fluorescence (Ex_440_ and Em_480_) was measured with a POLARstar Omega plate reader (BMG Labtech, Ortenberg, Germany).

### Generation and quantification of soluble protein aggregates

The procedure used to produce soluble aggregates of Aβ, α-synuclein and tau from fibrils was identical for each protein. End stage fibrils were pelleted by ultracentrifugation at 100,000×*g* for 1 h, and 90% of the supernatant was removed and retained for measurement by ELISA. The pellet was washed twice by resuspension in sterile PBS and centrifugation at 100,000×*g* for 1 h, and the final pellet was resuspended in sterile PBS. One-quarter of the washed fibrils were retained as fibril stocks, and the other three-quarters were immersion sonicated at 10 kHz for 10 × 5 s bursts with 15-s rests between bursts. Thereafter, the sonicated material was centrifuged at 16,000×*g* and 4 °C for 10 min, and 90% of the soluble supernatant was collected, aliquoted and stored frozen at – 80 °C until use.

Since SPAs scatter light and may not bind BCA uniformly it is not possible to use conventional assays to determine protein concentrations. Instead, we used ELISAs to measure the starting monomer concentration and the amount of monomer left in solution after sedimenting fibrils. We then used these values to calculate the per cent monomer incorporated into fibrils and hence, the molar amount of protein in fibrils, i.e. $100 - \left( {\frac{{\left[ {\text{initial monomer}} \right]{ } - { }\left[ {\text{monomer at end stage}} \right]}}{{\left[ {\text{initial monomer}} \right]}} x 100} \right)$. Across three independent experiments, we found that ~ 98% of starting Aβ was incorporated into fibrils, whereas ~ 78% of αSyn and ~ 77% of tau were incorporated into fibrils. Using this information, we estimated the molar concentration of the resulting SPA preparations.

### Sedimentation-ThT assays

To monitor ThT binding of fractions produced through the soluble aggregate generation process, portions of samples produced at each step were retained for end-point ThT assays. These fractions were: B, aggregation buffer alone; M, freshly prepared monomeric protein; N, neat end-stage fibrillary material; S, high-speed supernatants obtained after pelleting fibrils; P, high-speed fibril pellet; and sonicated soluble material. Protein concentrations and ratios to ThT were identical to the continuous or end-point assays used for each protein’s primary aggregation reactions.

### Solid-phase PrP binding assays

Three separate microtiter assays were employed: (i) an indirect ELISA to quantify binding of anti-PrP mAbs to recombinant PrP, (ii) a modified indirect ELISA to measure binding of forms of Aβ, α-synuclein, tau and BSA to recombinant PrP and (iii) a displacement ELISA to measure mAb-mediated inhibition of protein binding to PrP. The same clear, half-area, high-binding microtiter plates (#675061; Greiner Bio-One, Monroe, NC) were used for all assays, and EC_50_/IC_50_s were calculated using GraphPad Prism 7.1. All plates were developed with 50 µL/well 3,3′,5,5′-Tetramethylbenzidine (TMB; ThermoFisher, Waltham, MA), stopped by the addition of 50 µL/well 1 M H_2_SO_4_ and read at 450 nm using a SpectraMax plate reader (Molecular Devices, Sunnyvale, CA). For dilutions of antibodies used, please see Supplementary Table 2.

#### Antibody binding ELISAs

Plates were coated with 30 µL/well of 0.5 µM PrP_23–231_, PrP_91–231_, or PrP_119–231_ and incubated for 1 h at 37 °C and 300 RPM. Plates were washed three times with 100 µL/well PBS + 0.05% Tween-20 (PBST) and blocked with 100 µL/well of 2% BSA at room temperature (RT) for 2 h and 300 RPM. Plates were washed three times with 100 µL/well PBST before 30 µL/well of different anti-PrP mAbs diluted in PBST + 0.5% BSA were added to the plates and incubated at RT for 1 h and 300 RPM. Plates were again washed three times with 100 µL/well PBST and 30 µL/well horseradish peroxidase (HRP)-conjugated secondary antibodies (diluted 1:15,000 in PBST + 1% BSA) were added and incubated at RT for 1 h and 300 RPM. After 3 × 100 µL/well washes with PBST, plates were developed.

#### PrP-binding ELISAs

Plates were coated with 30 µL/well of 0.5 µM PrP_23–231_, PrP_91–231_, or PrP_119–231_ and incubated for 1 h at 37 °C and 300 RPM. Plates were washed three times with 100 µL/well PBS + 0.05% Tween-20 (PBST) and blocked with 100 µL/well of SuperBlock PBS (ThermoFisher, Waltham, MA) at room temperature (RT) for 2 h and 300 RPM. Plates were washed three times with 100 µL/well PBST before 30 µL/well of different proteins diluted in PBST + 0.5% BSA were added to the plates and incubated at RT for 1 h and 300 RPM. Plates were washed, and bound Aβ, αSyn, tau were detected with 30 µL/well 3D6, 2F12 and HJ8.5, respectively, diluted in PBST + 0.5% BSA at RT for 1 h and 300 RPM. Thereafter, bound antibodies were detected with 30 µL/well horseradish peroxidase (HRP)-conjugated secondary antibodies (diluted 1:15,000 in PBST + 1% BSA) at RT for 1 h and 300 RPM. After 5 × 100 µL/well washes with PBST, plates were developed.

#### Displacement ELISAs

Plates were coated with 30 µL/well of 0.5 µM PrP_23–231_ and incubated for 1 h at 37 °C and 300 RPM. Plates were washed three times with 100 µL/well PBS + 0.05% Tween-20 (PBST) and blocked with 100 µL/well of SuperBlock PBS (ThermoFisher, Waltham, MA) at room temperature (RT) for 2 h and 300 RPM. Plates were washed three times with 100 µL/well PBST before 30 µL/well of different anti-PrP mAbs diluted in PBST + 0.5% BSA were added to the plates and incubated at RT for 1 h and 300 RPM. After washing, plates were incubated with 156 nM Aβ, αSyn or tau and incubated at RT for 1 h and 300 RPM, washed, and bound Aβ, αSyn or tau detected with HRP-conjugated 3D6, 2F12 and HJ8.5, respectively, diluted in PBST + 0.5% BSA at RT for 1 h and 300 RPM. Thereafter, bound antibodies were detected with 30 µL/ well of streptavidin-HRP (R&D Systems, Minneapolis, MN) diluted 1:200 in PBST + 0.5% BSA at RT for 1 h and 300 RPM. After 5 × 100 µL/well washes with PBST, plates were developed.

### Negative contrast electron microscopy and quantification

Samples were stained and visualized essentially as described previously [10, 90]. Aliquots of freshly thawed protein samples were adsorbed (10 μl) neat onto formvar-coated copper grids (Electron Microscopy Sciences, Hatfield, PA) for 1 min before 10 μl of 0.25% glutaraldehyde (ThermoFisher, Waltham, MA) was added and incubated for 1 min. Thereafter, grids were wicked dry using qualitative filter paper (VWR, Radnor, PA), washed twice with 10 μl MilliQ water and then stained with 1% uranyl acetate for 2 min. Grids were wicked dry as above and allowed to air dry for at least 10 min, stored at room temperature and then examined using a 1200EX microscope (JEOL).

For measurement of soluble protein aggregates, at least three grids per protein were mounted, and at least three images per protein were analyzed. Length, defined as the long axis of each fragment measured, was measured using FIJI [77] and binned into 8 nm segments [66] using the scale bar as a standard (2.58 pixels/nm). Particles were excluded from analysis if they were on the border of the image or did not have clearly defined edges (i.e. due to a clustering). All particles meeting these criteria within the image field of view were also analyzed for width, defined as the short axis of each measured fragment.

### Animals

All procedures were performed in accordance with the National Institutes of Health Policy on the Use of Animals in Research and were approved by the Harvard Medical School Standing Committee on Animals. Founder *Prnp*^*−/−*^ mice [13] were from DAH’s colony. Single nucleotide polymorphism genotype scanning indicated *Prnp*^*−/−*^ mice were on a background of 98.13% C57BL/6J (The Jackson Laboratory, Bar Harbor, ME). Prior to expansion of the DMW colony *Prnp*^*−/−*^ mice were back-crossed with C57BL/6J mice (The Jackson Laboratory, Bar Harbor, ME) to generate hemizygotes that were then interbred to create wild-type (WT) and *Prnp*^*−/−*^ littermates. For each experiment, mice were genotyped by PCR before use. Animals were housed in a room with a 12 h light/dark circadian cycle with ad libitum access to food and water.

### Mouse primary neuronal cultures

Primary neurons were prepared as described previously [46], with modification. All media and solutions were 0.22 µm sterile filtered. Cortices were explanted from embryonic day 15–18 pups in ice-cold Dissection Buffer A (DSA: Phenol red-free HBSS with Ca^2+^ and Mg^2+^, 1 mM sodium pyruvate, 10 mM HEPES, pH 7.4). Tissue was transferred to ice-cold Dissection Buffer B (DSB: HBSS without Ca^2+^ and Mg^2+^, 1 mM sodium pyruvate, 10 mM HEPES, pH 7.4), carefully washed three times and microdissected free of meninges. Thereafter, cortices were partially dissociated in warm DSB with 0.125% trypsin–EDTA for 16 min at room temperature with gentle inversion every 4 min. Cortices were then rinsed three times in warm Neurobasal medium (NBM: Neurobasal, 1X GlutaMax-I, 1X B27 supplement). Complete dissociation was performed manually using a series of increasingly smaller pipette tips (1 mL tip, 200 μl tip, followed by a series of fire polished glass pipettes) followed by an incubation period of 15 min at 37 °C. Suspensions were strained through a 40-μM nylon cell strainer (Corning Life Sciences, Corning, NY) and intact cells separated from debris by centrifugation at 200 × *g* for 5 min. Cell pellets were resuspended in NBM, counted using a trypan blue-based automated cell counter (Bio-Rad, Hercules, CA) and plated on poly-l-lysine coated plates (MilliporeSigma, St. Louis, MO) at densities of 1 × 10^4^ or 1 × 10^6^ cells/well in 96-well and 6-well plates, respectively. Four days after plating, half the media was replaced with NBM containing 10 μm 5-fluoro-2′-deoxyuridine (MilliporeSigma, St. Louis, MO) to reduce proliferation of dividing cells. Cultures were kept in a humidified incubator at 37 °C with 5% CO_2_ and were supplemented once per week with fresh NBM. Neurons were cultured for 20–22 days prior to experimentation.

### Brain slice preparation

Two- to three-month-old male and female animals were deeply anesthetized with isoflurane and decapitated. Brains were excised and immediately immersed in ice-cold (0–4 °C) artificial cerebrospinal fluid (aCSF) comprised of (in mM): 124 NaCl, 3 KCl, 2.4 CaCl_2_, 2 MgSO_4_·7H_2_O, 1.25 NaH_2_PO_4_, 26 NaHCO_3_ and 10 d-glucose and was equilibrated with 95% O_2_ and 5% CO_2_, pH 7.4, 310 mOsm. Coronal brain slices (300 µm), including hippocampus were prepared using a Leica VT1000 S vibratome (Leica Biosystems Inc, Buffalo Grove, IL), transferred to an interface chamber and incubated at 34 ± 5 °C for 20 min, then kept at room temperature for 1 h before recording [90].

### Microelectrode array measurement of long-term potentiation (LTP)

A medium-throughput 64-channel MED64-Quad II system (Alpha MED Scientific, Osaka, Japan) was used for extracellular field potential recordings. Before each experiment, the surface of the MED64-Quad II probe was treated with 0.7 mL 0.1% polyethyleneimine (PEI) in 25 mM borate buffer (pH 8.4) overnight at room temperature. The following day, PEI was aspirated, the probe was rinsed with distilled water 4 times, covered with 1 mL distilled water and stored at 4 °C until use. Thereafter, brain slices were transferred to the MED probe 16 (2 × 8) and the electrodes were positioned in the stratum radiatum of CA1 under the guidance of inverted phase contrast microscope (Nikon TMS-F, Nikon Instruments Inc, Melville, NY). Reference images were taken by ISCapture using a digital camera (AmScope) connected to the microscope. Once the slice settled, a fine-mesh and a slice anchor (Warner Instruments, Harvard Bioscience Inc.) were carefully positioned to ensure slice stabilization during recording. The MED probe was then inserted into the MED connector (MED64 ThermoBase II, MED-CPB02) and a perfusion cap was used to circulate oxygenated aCSF (maintained at 30 °C) into the probe and prevent the slices from drying. Perfusion was controlled by a MED64 pump (input rate of 1.6 mL/min) while humidified oxygen entered the probe at 0.5 L/min. A total of four brain slices per experiment were placed on the 4 MED16 probes and, after 10 min of recovery, 1 of 16 (2 × 8) planar microelectrodes was selected as the stimulation electrode. Field excitatory postsynaptic potentials (fEPSPs) were evoked by a monopolar, biphasic constant current pulse (0.2 ms in duration) generated by the data acquisition software (Mobius, Alpha-Med Scientific). The electrode along the Schaffer collaterals with the best fEPSPs was chosen as recording electrode. Next, the input–output (I/O) curve was measured and the baseline stimulation intensity was set to 40% of maximum fEPSPs slope. During baseline recording, stimuli were delivered to 4 slices with 5-s intervals. After 30 min, LTP was induced by theta burst stimulation (TBS, 10 bursts at 5 Hz, 4 pulses at 100 Hz for each burst) three times with 4.25-s intervals. After TBS, the baseline stimulus was given to four slices repeatedly with 5-s intervals for another hour. Signals were amplified by the MED64 Main Amplifier (MED-A64MD1A) and MED64 Head Amplifier (MED-A64HE1S), then digitized at a 10-kHz sampling rate. The digitized data were exported for offline analysis. Within each measurement, one slice was used for aCSF control while the other three slices were treated with different concentration of SPAs. Each set of four slices were from a single animal and between three and five animals were used for each SPA experiment.

### Immunocytochemistry and quantification

Mouse primary neurons (MPNs) and induced neurons (iNs) seeded in clear bottom, black wall 96-well plates (#655090; Greiner Bio-One, Monroe, NC) were immunostained using a modified serial-permeabilized protocol [33]. All buffers and reagents were prepared fresh and 0.22 µm filtered prior to use. Cells were washed three times with warm aCSF and fixed with ice-cold 4% paraformaldehyde/4% sucrose in PBS for 10 min at 4 °C. Thereafter, plates were washed once with PBS, quenched with 0.1 M glycine in PBS for 10 min at room temperature, washed three times with PBS and stored at 4 °C overnight. After fixing, all subsequent steps were performed on a shaker set to speed ‘2’. The following day, plates were warmed to room temperature for 30 min and processed one of two ways: (i) for total cellular staining, cells were permeabilized with 0.25% Triton X-100 in PBS for 5 min prior to blocking, or (ii) for surface staining, cells were immediately blocked without the permeabilization step. Blocking was done with 3% BSA in PBS for 2 h before plates were washed three times with PBS and incubated for 2 h with primary antibodies (see Supplementary Table 2 for antibodies and dilutions) diluted in 3% BSA/PBS. Thereafter, plates were washed 6 × 5 min with 0.05% PBST, and plates that had not been previously permeabilized were treated with 0.25% Triton X-100 in PBS for 5 min to unmask intracellular epitopes, incubated with primary antibodies (as above) against structural markers (Supplementary Table 2) and washed 6 × 5 min with 0.05% PBST. Bound antibodies were detected by the addition of the appropriate Alexa Fluor conjugated secondary antibodies (diluted 1:500 in 3% PBST/BSA; ThermoFisher, Waltham, MA) for 1 hour and plates were washed 6 × 5 min with 0.05% PBST, incubated for 5 min with DAPI (1 µg/mL; ThermoFisher, Waltham, MA) in PBS, washed a further three times with PBS and imaged using a GE InCell 2200. The acquisition settings were as follows: objective, Nikon 20X/0.45 Pan-Fluorescent CFI/60; channels, DAPI, FITC_511, Cy3, Cy5; binning, 1 × 1; polychroic, QUAD1; laser autofocus, 10%. All images were flat-field corrected with 2-D deconvolution, and quantification of puncta was performed using a custom, automated FIJI macro.

### On-cell western blot

Binding of soluble aggregates to MPNs was analyzed using a modified on-cell Western blot protocol. All buffers and reagents were prepared fresh and 0.22 µm filtered prior to use and infrared dye-compatible reagents were from LI-COR Biosciences (Lincoln, NE). On DIV 20–22, cells seeded at 1 × 10^4^ cells/well in 96-well clear bottom, black wall 96-well plates (#655090; Greiner Bio-One, Monroe, NC) were treated with soluble protein aggregates for 2 h at 37 °C. Cells were washed three times with warm aCSF and fixed with ice-cold 4% paraformaldehyde/4% sucrose in PBS for 10 min at 4 °C. Thereafter, plates were washed once with PBS, quenched with 0.1 M glycine in PBS for 10 min at room temperature, washed three times with PBS and stored at 4 °C overnight. After fixing, all subsequent steps were performed on a shaker set to speed ‘2’. The following day, plates were warmed to room temperature for 30 min and blocked in 100 µL/well LI-COR Blocking Buffer for 2 h at RT. Thereafter, primary antibodies to surface antigens were diluted in LI-COR Blocking buffer and incubated for 2 h at RT. Cells were then washed with PBS, permeabilized by the addition of with 0.25% Triton X-100 in PBS for 5 min, incubated with primary antibodies against structural markers (Supplementary Table 2) and washed 6 × 5 min with 0.05% PBST. IR-labeled secondary antibodies (diluted 1:1000 in LI-COR Blocking Buffer + 0.1% Tween-20) were added for 1 h at RT before plates were washed extensively with PBST and imaged using a LI-COR Odyssey CLx infrared imaging system.

### SDS-PAGE, coomassie staining and western blotting

Mouse primary neurons and iNs were seeded in 6-well-plates at 1 × 10^6^ or 5 × 10^5^ cells/well, respectively, washed twice with warm aCSF, scraped into 1% CHAPS lysis buffer (1% CHAPS, 30 mM Tris–HCl, 150 mM NaCl pH 7.5 + protease and phosphatase inhibitors) and incubated on ice for 10 min. Suspensions were homogenized on ice with 20 strokes of a motorized Teflon pestle (Argos Technologies, Vernon Hills, IL), freeze-thawed once, incubated a further 10 min on ice and centrifuged 10,000×*g* for 15 min. Postnuclear supernatants were retained and protein concentrations determined by A_280_ (NanoDrop 2000, ThermoFisher, Waltham, MA). Ten to thirty µg lysate or 0.25 to 1 µg recombinant/synthetic protein was mixed with 4X LDS-phenol red sample buffer, boiled and electrophoresed on 17- or 26-well precast 4–12% Bis–Tris gels (ThermoFisher, Waltham, MA). For Coomassie staining of purified proteins, gels were rinsed briefly with MilliQ, fixed in 50% methanol, 10% glacial acetic acid, washed 2 × 5 min in MilliQ and stained with GelCode Blue (ThermoFisher, Waltham, MA) for 1 h at room temperature. Gels were destained with frequent washes in MilliQ and imaged using a LI-COR Odyssey CLx infrared imaging system (all LI-COR, Lincoln, NE). For Western blotting, proteins were transferred onto 0.2 µm nitrocellulose at 400 mA and 4 °C for 2 h, and total protein was visualized by REVERT stain. Membranes were then blocked for 1 h with Odyssey Blocking Buffer, incubated with primary antibodies (Supplementary Table 2) overnight at 4 °C, washed 6 × 10 min with PBST, incubated with IR-dye conjugated secondary antibodies (1:17,000), washed 6 × 5 min with PBST and bands were visualized using a LI-COR Odyssey CLx infrared imaging system (all LI-COR, Lincoln, NE). In some cases, densitometry was performed using FIJI (Supplementary Figure 4) by normalizing the optical densities of PrP, tau and β-III-tubulin bands to those for GAPDH [77].

### Production of induced neurons (iNs) from human induced pluripotent cells (iPSCs)

The YZ1 iPSC line [95] was used to prepare neurogenin 2 (Ngn2)-induced human neurons [96] as described previously [41, 45]. On iN day 4, cells were plated at 4000 cells/well on Matrigel (Corning Life Sciences, Corning, NY)-coated plates (#655090; Greiner Bio-One, Monroe, NC) and maintained until iN day 21, a time point when iNs are fully mature (Supplementary Figure 5A).

### CRISPR iPSC editing

Single guide RNA (sgRNA) sequences were designed as previously reported [76] and cloned into the pXPR_003 plasmid (Addgene #52963) by PCR. Healthy control YZ1.4 iPSCs (1 × 10^5^) were plated on growth factor-reduced Matrigel (Corning Life Sciences, Corning, NY) and cultured in StemFlex medium. The following day, cells were co-transfected with SpCas9 (pXPR_BRD111Cas9v2; Addgene #78166) and *PRNP* sgRNA or empty plasmids using Lipofectamine 2000 (ThermoFisher, Waltham, MA). Two days post-transfection, iPSCs were selected with puromycin (5 µg/mL) and blasticidin (4 µg/mL). Thereafter, genomic DNA was extracted from a subset of cells and editing was monitored by mismatch assay (GeneArt Genomic Cleavage Detection Kit, ThermoFisher, Waltham, MA). Limited dilution subcloning was used to isolate edited iPSCs, and monoclonal lines were examined by PCR-sequencing around the edited region. Hit clones were expanded, differentiated into iNs and cultured until iN D21, at which point cells were harvested for Western blot analysis of PrP expression.

### Quantitative real-time PCR

RNA was extracted from iPSCs and iNs with TRIzol, reverse-transcribed and complementary DNA (cDNA) was used for quantitative real-time PCR (qPCR) using Fast SYBR Green Master Mix on a ViiA 7 system (all ThermoFisher, Waltham, MA). Data were normalized to glyceraldehyde 3-phosphate dehydrogenase (GAPDH) expression using the ΔΔCT method [56].

### Live-cell imaging and neuritotoxicity assays

On iN DIV21 or when primary mouse neurons were 20–22 DIV, plates were transferred to an IncuCyte Zoom live-cell imaging instrument (Essen Bioscience, Ann Arbor, MI) and images collected every 2 h for a total of 6 h. These three image sets were used to establish ‘before treatment’ baseline neurite length measurements. Immediately thereafter, ¾ the culture medium was removed (leaving ~ 50 µL) and recombinant protein preparations were diluted into 50 µL fresh NBM and added to cells (three technical replicates per protein per plate, three experimental replicates in total). When human brain extracts were tested, ½ the culture medium was removed (leaving ~ 100 µL) and 25 µL desalted extracts (see ‘*Preparation of Human Brain Extracts’* below) were diluted with 75 µL fresh NBM and added to cells. Phase-contrast images were collected from four fields per well every 2 h (for a total of 96 h) and analyzed using the IncuCyte Zoom 2016A NeuroTrack analysis platform (Essen Bioscience, Ann Arbor, MI) to define neurites and somas. Total neurite length (in mm) was quantified and normalized to the average value measured during the 6-h period prior to sample addition.

### Preparation of human brain extracts

Human specimens used for biochemical experiments were obtained from the Massachusetts ADRC Neuropathology Core, Massachusetts General Hospital and used in accordance with the Partners Institutional Review Board (Protocol: Walsh BWH 2011). Frozen cortical tissues were obtained from seven cases, two each who died with AD, DLB and FTD and one individual who died free of any signs of neurodegeneration (Supplementary Table 1). All diseased cases met current post-mortem and clinical diagnostic criteria for the relevant disorder. Aqueous extracts were prepared as described previously [41]. Briefly, grey matter was dissected free of white matter/vasculature and homogenized in five volumes of ice-cold base artificial cerebrospinal fluid (aCSF-B; 124 mM NaCl, 2.8 mM KCl, 1.25 mM NaH_2_PO_4_, 26 mM NaHCO_3_, pH 7.4) plus protease inhibitors (5 mM ethylenediaminetetraacetic acid (EDTA), 1 mM ethyleneglycoltetraacetic acid (EGTA), 5 μg/mL leupeptin, 5 μg/mL aprotinin, 2 μg/mL pepstatin, 120 μg/mL Pefabloc and 5 mM NaF) with 25 strokes of a mechanical Teflon-glass Dounce homogenizer (Fisher, Ottawa, Canada). Resulting homogenates (20% w/v) were centrifuged at 200,000×*g* and 4 °C for 110 min in a SW41 Ti rotor (Beckman Coulter, Fullerton, CA). The upper 80% of the supernatant was removed and dialyzed against 100-fold excess of fresh aCSF-B at 4 °C using 2 kDa MWCO Slide-A-Lyzer cassettes (ThermoFisher, Waltham, MA). Buffer was changed three times over a 72-h period before dialyzed extracts were aliquoted and stored at – 80 °C until use.

### Solid-phase sandwich immunoassays

*Aβx-42 MSD Assays:* Analysis of Aβ in soluble brain extracts and in supernatants from end-stage Aβ_1–42_ fibril harvests were performed using monomer-preferring Aβ_*x*−42_ immunoassays [60] employing m266 for capture and biotinylated 21F12 for detection (Supplementary Table 2). Assays were performed using the Meso Scale Discovery (MSD, Rockville, MD) platform and a Sector imager to analyze plates. The lower limit of quantitation (LLoQ), determined by calculating the average + 9 standard errors and 100 ± 20% recovery for each standard, was 39.06 pg/mL.

#### Tau enzyme-linked immunosorbent assays (ELISAs)

Analysis of tau in soluble brain extracts was performed using a mid-region ELISA (BT2 as capture, Tau5 for detection), while tau in supernatants from end stage fibril harvests was measured using an N-terminal ELISA (Tau12 as capture, Tau5 for detection). Assays were performed exactly as described [37, 42] and details regarding the antibodies employed are listed in Supplementary Table 2. Standard curves were fitted to a five-parameter logistic function with 1/Y^2^ relative weighting using MasterPlex Software (MiraiBio). LLoQs were calculated as described for the MSD assays and were 31.25 pg/mL and 15.625 pg/mL for mid-region and N-terminal assays, respectively.

#### α-synuclein ELISA

α-synuclein was measured in soluble brain extracts and in supernatants from end stage fibril harvests using a C-terminal-directed assay. Black half-area, high-binding plates (#655090; Greiner Bio-One, Monroe, NC) were coated with 7 µg/mL SOY1 in TBS for 1 h at 37 °C and 300 RPM. Plates were then washed three times with 100 µL TBS + 0.05% Tween-20 (TBST) prior to blocking in 100 µL TBS containing 3% BSA for 2 h at RT and 300 RPM. Plates were washed three times with 100 µL TBST before 25 µL samples (diluted 1:500 for brain extracts and 1:400 k for fibril supernatants in TBS containing 1% BSA) and standards were applied in triplicate and agitated for 16 h at 4 °C. Next, 25 µL alkaline phosphatase conjugated 2F12 (diluted 1:250 in TBST containing 1% BSA) was added directly to the plates without washing and incubated for 1 h at RT and 300 rpm. Finally, plates were washed three times with 100 µL TBST before 50 µL Tropix Sapphire II (Applied Biosystems) detection reagent was added and incubated for 30 min at RT and 300 RPM. Standard curves were fitted as described for the tau ELISAs LLoQs (62.5 pg/mL) were calculated as described for both the Aβ MSD and tau assays.

### Immunoprecipitation/western blotting from human brain extracts

#### Aβ

Freshly thawed extracts (1 mL) from brain AD1 were pre-cleared of nonspecific IgG with 15 μl protein A sepharose (PAS) beads for 1 h at 4 °C. PAS beads were sedimented (4000 × *g* for 5 min) and the supernatant divided into 0.5 mL aliquots. Each aliquot was incubated with 10 µL of AW7 (Supplementary Table 2) or preimmune serum (PIS; negative control) and 15 μl PAS beads overnight at 4 °C with agitation. Complexes were collected by centrifugation and washed as previously described [90]. To effectively deplete Aβ, extracts were subjected to three serial rounds of IP and a final PAS IP alone to remove unbound IgG. The immunoprecipitated (IP’d) Aβ was eluted by boiling in 15 μl of 2X sample buffer (50 mM Tris, 2% w/v SDS, 12% v/v glycerol with 0.01% phenol red) and electrophoresed on hand poured, 15-well 16% polyacrylamide tris–tricine gels. Synthetic Aβ_1–42_ was included as a loading control and protein transferred onto 0.2 µm nitrocellulose at 400 mA and 4 °C for 2 h. Blots were microwaved in PBS and Aβ detected using the anti-Aβ40 and anti-Aβ42 antibodies, 2G3 and 21F12, and bands visualized using a Li-COR Odyssey CLx infrared imaging system (Li-COR, Lincoln, NE).

#### α-synuclein

Aqueous brain extract was depleted of α-synuclein using the antibody 2F12 (Supplementary Fig. 9). Freshly thawed extract (1 mL) from DLB1 was first pre-cleared of non-specific IgG with 20 μl protein G agarose (PGA; Roche Life Sciences, Madison, WI) beads for 1 h at 4 °C. PGA beads were removed by centrifugation (4000×*g* for 5 min), the supernatant transferred to fresh tubes and incubated with 100 µg/mL 2F12 or 46–4 (isotype control, Supplementary Table 2) overnight at 4 °C with agitation. The following day, 2 mL 2F12- or 46–4-coupled brain extracts were manually injected over 1 mL HiTrap Protein G HP columns (GE Life Sciences, Marlborough, MA) that had been equilibrated with 10 CV aCSF-B. Half-millilitre fractions were collected from the flow through (ID fraction), protein concentrations were measured using bicinchionic acid (BCA) assays (ThermoFisher, Waltham, MA) and the four peak fractions (#3–6) pooled. Then, using a peristaltic pump, columns were washed with 20 CV aCSF-B before bound antibody-antigen complexes were eluted in 5 CV 0.1 M glycine, pH 2.7. Again, half-millilitre fractions were collected from the elution (IP fraction) and immediately neutralized with 40 µL 1 M Tris (unbuffered; pH 10–11). Protein concentrations from the IP fraction were determined by A_280_ (NanoDrop 2000, ThermoFisher, Waltham, MA), and the peak fraction (#3) was buffer-exchanged into 50 mM (NH_4_)HCO_3_, pH 8.5 using 7 kDa Zeba desalting columns (ThermoFisher, Waltham, MA). Samples were then lyophilized, resuspended and boiled in 40 µL 1X lithium dodecyl sulfate (LDS)-phenol red sample buffer (26 mM Tris–HCl, 35 mM Trizma base, 0.5% LDS, 2.5% glycerol, 0.125 mM EDTA, 0.04 mM phenol red). For Western blotting, 10 µL and 4 µL ID and IP fractions, respectively, were electrophoresed on precast 4–12% Bis–Tris gels (ThermoFisher, Waltham, MA) with recombinant α-synuclein included as a loading control. Proteins were transferred onto 0.2 µm nitrocellulose at 400 mA and 4 °C for 2 h, α-synuclein was detected using antibody C-20 (Supplementary Table 2) and bands were visualized using a LI-COR Odyssey CLx infrared imaging system (LI-COR, Lincoln, NE).

#### Tau

Tau was depleted from human brain extracts essentially as described for [67]. Freshly thawed extracts (1 mL) from PiD brain were first pre-cleared of endogenous IgG with 20 μl PGA beads for 1 h at 4 °C. PGA beads were sedimented out of solution (4000 × *g* for 5 min) and the supernatant divided into 0.5 mL aliquots. Each aliquot was treated with 10 µg Tau5 and 15 µL PGA, or 10 µg 46–4 (isotype control) and 15 µL PGA and incubated with agitation overnight at 4 °C. Complexes were collected by centrifugation and washed 3 × 10 min in 1 mL PBS. To effectively deplete tau, extracts were subjected to three serial rounds of IP and a final PGA IP alone to remove unbound IgG. The IP’d tau was eluted by boiling in 15 μl of 2X LDS-phenol red sample buffer. For Western blotting, 5 µL each ID and IP fractions were electrophoresed on precast 4–12% Bis–Tris gels (ThermoFisher, Waltham, MA) with recombinant hTau40 included as a loading control. Proteins were transferred onto 0.2 µm nitrocellulose at 400 mA and 4 °C for 2 h, tau was detected using antibody K9JA (Supplementary Table 2) and bands were visualized using a LI-COR Odyssey CLx infrared imaging system (LI-COR, Lincoln, NE).

### Array tomography microscopy

Post-mortem human brain tissue was obtained from the Edinburgh Brain and Tissue Bank and the Massachusetts General Hospital Alzheimer’s Disease Research Center brain bank with full ethical approval (TSJ AMREC ethical approval number 15-HV-016). The use of post-mortem human tissue for scientific purposes has been approved by the Edinburgh Brain Bank ethics committee and the ACCORD (Academic and Clinical Central Office for Research and Development, collective office of the University of Edinburgh and the National Health Service Lothian) committee for medical ethics AMREC. The Edinburgh Brain Bank is funded by the Medical Research Council with approval from the Research Ethics Committee (REC), (11/ES/0022). Tissue obtained was donated by individuals and their families, with ethical and legal approval. The tissue is collected, processed and embedded for array tomography microscopy at autopsy as described previously [49]. Briefly, tissue from temporal cortex Brodmann area 20/21 was fixed in 4% paraformaldehyde for 2–3 h, dehydrated through an ethanol gradient and incubated overnight in LR White resin (Electron Microscopy Sciences) overnight at 53 °C. Samples were from cases with clinicopathological diagnoses of DLB (*n* = 3), AD, (*n* = 3) and FTD (*n* = 3) (Supplementary Table 1). In each case, a ribbon of at least ten 70 nm-thick consecutive sections was produced using a histo jumbo diamond knife (Diatom) on an ultracut microtome (Leica), mounted on glass coverslips and stained as described previously [49]. Briefly, ribbons were stained with DAPI and antibodies to PrP plus synaptophysin and Aβ, αSyn, or phosphorylated tau (pTau) (Supplementary Table 2). Images were acquired from the same region of the temporal cortex on ten consecutive serial sections using a Leica TCS8 confocal with a 63 × 1.4 NA oil objective. Laser and detector settings were set on a positive control human post-mortem brain section which contained plaques, tangles or Lewy bodies. Alexa Fluor 488, Cy3 or Alexa Fluor 647 were sequentially excited with the 488, 552 or 638 nm laser lines and imaged in 500 to 550 nm, 570 to 634 nm or 649 to 710 nm spectral windows, respectively. A 405-nm laser was used for DAPI visualization collecting images in a spectral range of 415 to 482 nm. Confocal images were aligned and segmented using a custom MATLAB algorithm (freely available at https://github.com/arraytomographyusers/Array_tomography_analysis_tool). 3D reconstructions and visualization were made with FIJI [77], Paraview [3] and Inkscape (https://www.inkscape.org).

## Results

### Soluble aggregates of Aβ, α-synuclein and tau bind to PrP

**Fig. 1:**
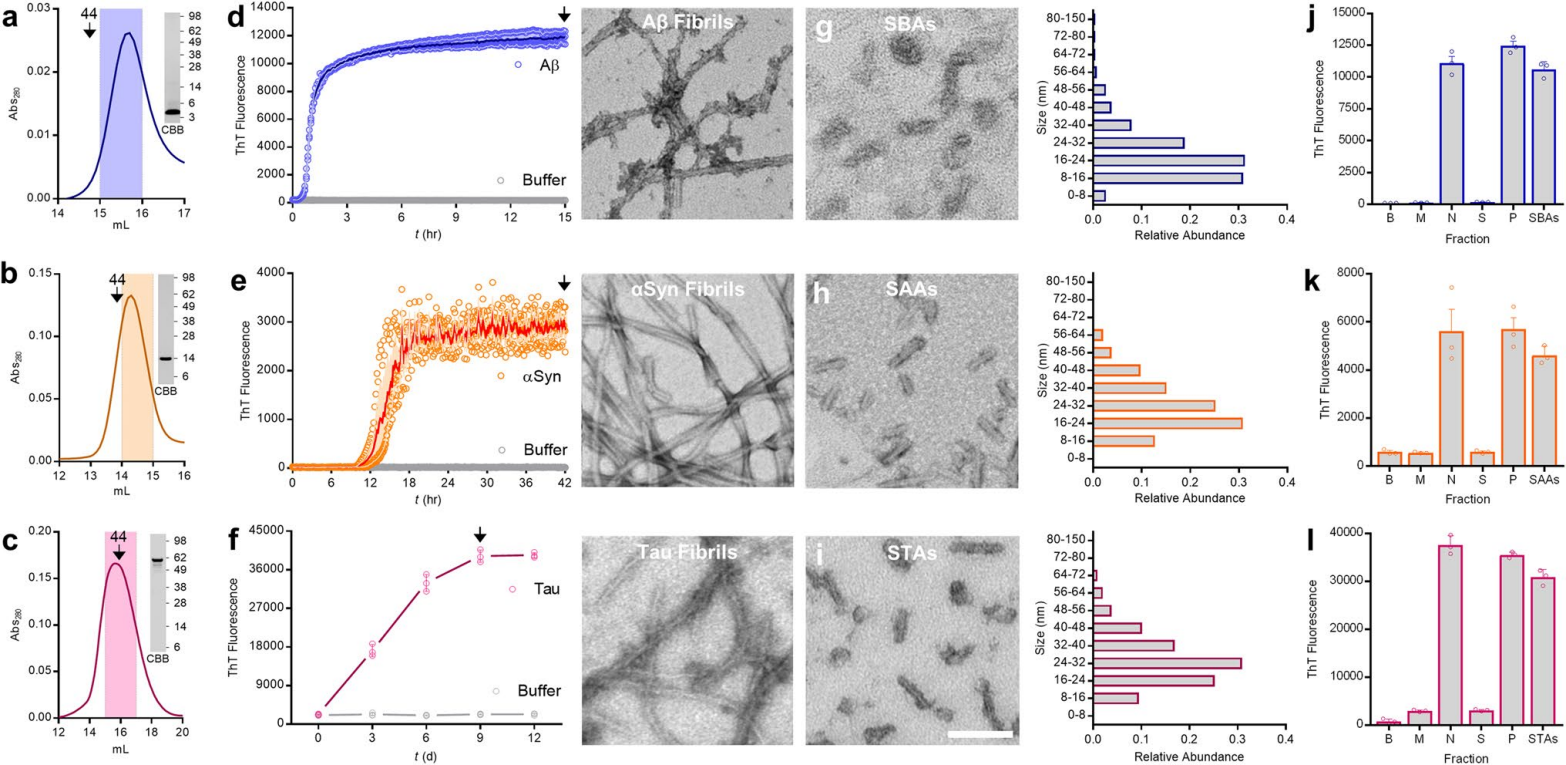
Preparation and characterization of soluble aggregates of Aβ, α-synuclein and tau. **a**–**c** Representative chromatograms depicting isolation of monomeric (**a**) Aβ_1–42,_ (**b**) α-synuclein (αSyn), and (**c**) tau. Downward arrows indicate elution of globular molecular weight standards, the shaded region indicates the fraction retained for aggregation experiments and the inset SDS-PAGE/coomassie (CBB) depicts migration of the purified monomers. **d–f** Freshly SEC-isolated monomer was diluted to 20 µM (Aβ_1-42_; **d**) and 35 µM (αSyn; **e**) and 50 µM (tau; **f**), combined with Thioflavin-T (ThT) and aggregation monitored until ThT signals plateaued (*T*_max_). Aβ and αSyn were aggregated without additives while tau was aggregated in the presence of 50 and 100 µM heparin and DTT, respectively. Binding of ThT to Aβ_1–42_ and αSyn was monitored continuously, whereas aliquots of tau were removed, mixed with ThT and assessed at 3-day intervals. In each case, identical reactions without protein (buffer) served as negative control. Fibrils were harvested at time points indicated by downward arrows, mounted on grids and representative negative-stain electron microscope (EM) micrographs of Aβ, αSyn and tau *T*_max_ fibrils are presented to the right of each graph**. g–i** Representative negative-stain EM micrographs of immersion sonicated soluble aggregates (Aβ SBAs, **g**; αSyn SAAs, **h**; tau STAs, **i**). Size distribution of SBAs, SAAs and STAs as determined by negative-stain EM are presented to the right of each micrograph. **j–l** Neat *T*_max_ fibrils (N), the supernatant of centrifuged fibrils (S), resuspended washed fibril pellets (P), and SBAs (**j**), SAAs (**k**), and STAs (**l**) were used for ThT binding. Buffer alone (B) and freshly isolated monomer (M) were included as negative controls. Scale bar in micrographs from **d–i** = 100 nm. Data in **d–f** and **j–l** are the mean ± SD, indicate three technical replicates and are representative of at least three independent experiments. Molecular weight markers (in kDa) are indicated to the right of the inset CBB gels in **a**, **b** and **c**

Extensive evidence indicates that soluble aggregates of synthetic Aβ bind to PrP. Most prior studies used ADDLs [19, 23, 31, 32, 66, 73, 84, 91, 93], a recipe-based preparation of Aβ_1–42_ which contains monomers, protofibrils and globular oligomers [39, 52, 66]. Here, we set out to determine if soluble aggregates of two other disease-linked proteins can also bind to PrP. Since the recipe used to generate ADDLs might not apply to other proteins, we first sought to establish protocols to enable generation of soluble aggregates of the three proteins of interest. Rather than try to trap intermediates of fibrillogenesis, we opted to use well-established, protein-specific conditions to form end-stage fibrillar aggregates [14, 38, 68] and then liberate soluble aggregates from these insoluble materials using a standardized sonication protocol. For each protein the process began by using size exclusion chromatography (SEC)-isolated monomers (Fig. 1a–c). Thereafter, protein-specific conditions were employed to produce amyloid fibrils (Fig. 1d–f). Thioflavin-T (ThT) binding was used to monitor aggregation and T_max_ fibrils produced in the absence of ThT were sedimented, washed to remove monomers, then resuspended and sonicated to produce soluble aggregates of Aβ (SBAs; Fig. 1g), αSyn (SAAs; Fig. 1h) and tau (STAs; Fig. 1i). EM analysis of SPAs revealed fragmented, protofibrillar-like species, the majority of which were 16–40 nm in length (Fig. 1g–i; histograms to the right), and SPAs retained > 80% of the ThT-binding capacity found in *T*_max_ fibrils (Fig. 1j–l).

**Fig. 2:**
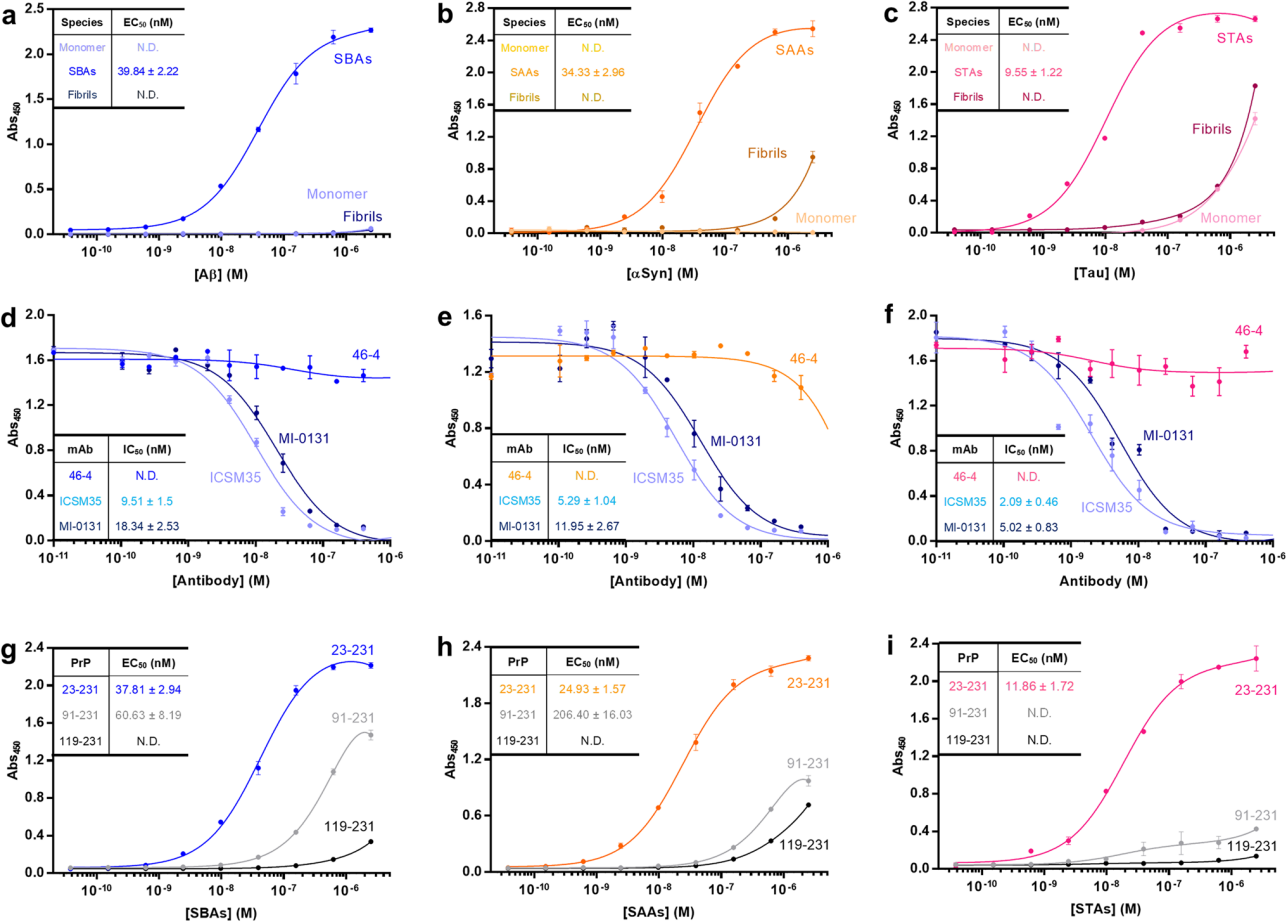
Soluble aggregates of Aβ, α-Synuclein and Tau bind to the N-terminus of PrP. **a–c** Binding of Aβ (**a**), αSyn (**b**) and tau (**c**) monomers, soluble aggregates and fibrils to immobilized PrP_23–231_ was assessed using an ELISA-like microtiter plate assay. Data shown are the mean ± SD from a single experiment, whereas inset EC_50s_ are from at least four independent experiments. **d–f** Binding of soluble protein aggregates to immobilized PrP_23–231_ can be inhibited by anti-PrP monoclonal antibodies (mAbs) to *Sites I* (MI-0131) and *II* (ICSM35), but not a nonspecific mAb (46–4). Data shown are the mean ± SD from a single experiment, whereas inset IC_50s_ are from at least four independent experiments. **g–i** To determine the regions of PrP involved in binding soluble protein aggregates, PrP_23–231,_ PrP_91–231,_ and PrP_119–231_ were immobilized and binding of SBAs (**g**), SAAs (**h**) and STAs (**i**) measured. Data shown are the mean ± SD from a single experiment, whereas inset EC_50s_ are from at least 4 independent experiments

The ability of different forms of Aβ, αSyn, and tau to bind recombinant PrP_23–231_ was assessed using a solid-phase assay. SBAs bound to PrP_23–231_ with an average EC_50_ of 38.9 nM, whereas neither Aβ monomers nor fibrils showed appreciable affinity for PrP (Fig. 2a). SAAs (Fig. 2b) and STAs (Fig. 2c) also bound to PrP in a saturable manner with average EC_50s_ of 34.3 nM and 9.5 nM, respectively. Monomeric and fibrillar forms of αSyn and tau exhibited weak or no affinity for PrP_23–231_. Thus, like Aβ, binding of αSyn and tau to PrP is aggregation state-specific. Interestingly, STAs bound more tightly to PrP than SAAs or SBAs. In contrast, soluble aggregates of bovine serum albumin (BSA) bound very weakly to PrP.

Binding of Aβ to PrP is mediated by two distinct sites within the N-terminal half of PrP (*Sites I* and *II*, Supplementary Fig. 1a) [19, 32, 53, 66]. To explore if binding of SAAs and STAs is mediated by the same sites on PrP, we tested whether mAbs directed to *Site I* (MI-0131) and *Site II* (ICSM35) (Supplementary Table 2) could prevent SPA binding. Both mAbs bound similarly well to PrP (Supplementary Figure 1e) and displaced SBAs to a comparable extent, with ICSM35 always slightly more effective (Fig. 2d). Interestingly, comparable IC_50_ trends were observed for SAAs (Fig. 2e; ICSM35 = 5.2 nM and MI-0131 = 11.9 nM) and STAs (Fig. 2f; ICSM35 = 2.0 nM and MI-0131 = 5.0 nM). Since antibodies (~ 150 kDa) are large relative to PrP (~ 28 kDa), we further investigated binding of SPAs to PrP using deletion constructs lacking most of *Site I* (PrP_91–231_) or both *Sites I* and *II* (PrP_119–231_) (Supplementary Figure 1b-d). As expected, SBAs did not bind PrP_119–231_ and weakly bound PrP_91–231_ (Fig. 2g). Similarly, SAAs and STAs showed little affinity for PrP_119–231_ and greatly diminished binding to PrP_91–231_ (Fig. 2h, i). Indeed, for STAs, deletion of *Site I* (PrP_91–231_) completely abolished binding (Fig. 2i). Collectively, deletion construct and mAb displacement experiments indicate that binding of SBAs, SAAs and STAs require *Sites I* and *II*, but the relative importance of these sites differs depending on the particular SPA.

#### SPAs bind to neurons in a saturable and PrP-dependent manner

**Fig. 3:**
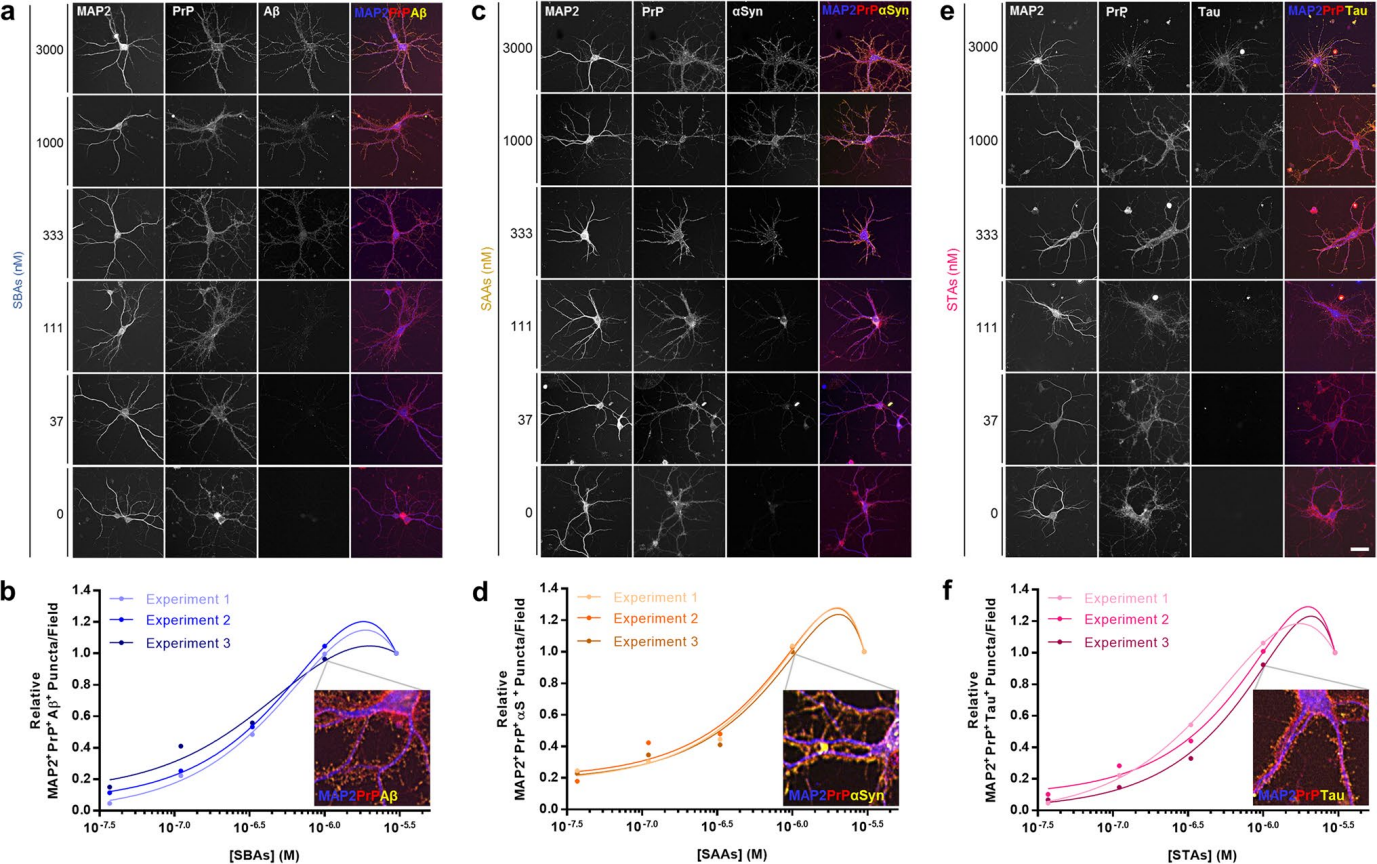
SBAs, SAAs and STAs bind to primary neurons in a dose-dependent and saturable manner. **a**, **c**, **e** Soluble protein aggregates were added to primary mouse neurons (MPNs) and binding assessed using serial-permeabilized immunocytochemistry. Representative images SBAs (**a**), SAAs (**c**) and STAs (**e**) are shown. Staining for MAP2, PrP and bound proteins are shown in blue, red and yellow, respectively. **b**, **d**, **f** Relative dose–response binding of SBAs (**a**), SAAs (**c**) and STAs (**e**) to MPNs was quantified using a custom FIJI macro that identified soluble protein aggregate puncta that were at least 50% PrP colocalized. Relative values were determined by normalizing binding signals to those obtained with the highest protein concentration analyzed (3 µM). Inset images depict enlarged triple-colocalization images for neurons treated with 1 μM SPAs (from **a**, **c** and **e**). Scale bar in **a**, **c** and **e** = 50 µm, and data in **b**, **d** and **f** represent three independent experiments with 60 images analyzed per experiment per dose

Next, we determined whether different forms of Aβ, αSyn and tau could bind to the surface of cortical neurons and whether binding depended on PrP. Mouse primary neurons (MPNs; Supplementary Fig. 2a) were incubated with monomers or SPAs for 2 h, washed extensively and stained using serial permeabilized immunocytochemistry (ICC)—a method which avoids detection of endogenous Aβ, αSyn and tau (Supplementary Figure 2b and c). Incubation of WT MPNs with SPAs revealed clear dose-dependent binding (Fig. 3a, c, e), whereas monomeric proteins, even when tested at 3000 nM, did not bind MPNs (Supplementary Figure 2d–f). Quantification of co-localized PrP-immunoreactive (IR) and SPAs-IR puncta confirmed that binding of SBAs (Fig. 3b), SAAs (Fig. 3d) and STAs (Fig. 3f) to MPNs was dose-dependent and saturable. Of note, we did not monitor binding of fibrils to MPNs because it is not possible to generate soluble preparations of fibrils and because fibrils can nonspecifically precipitate onto membranes.

**Fig. 4:**
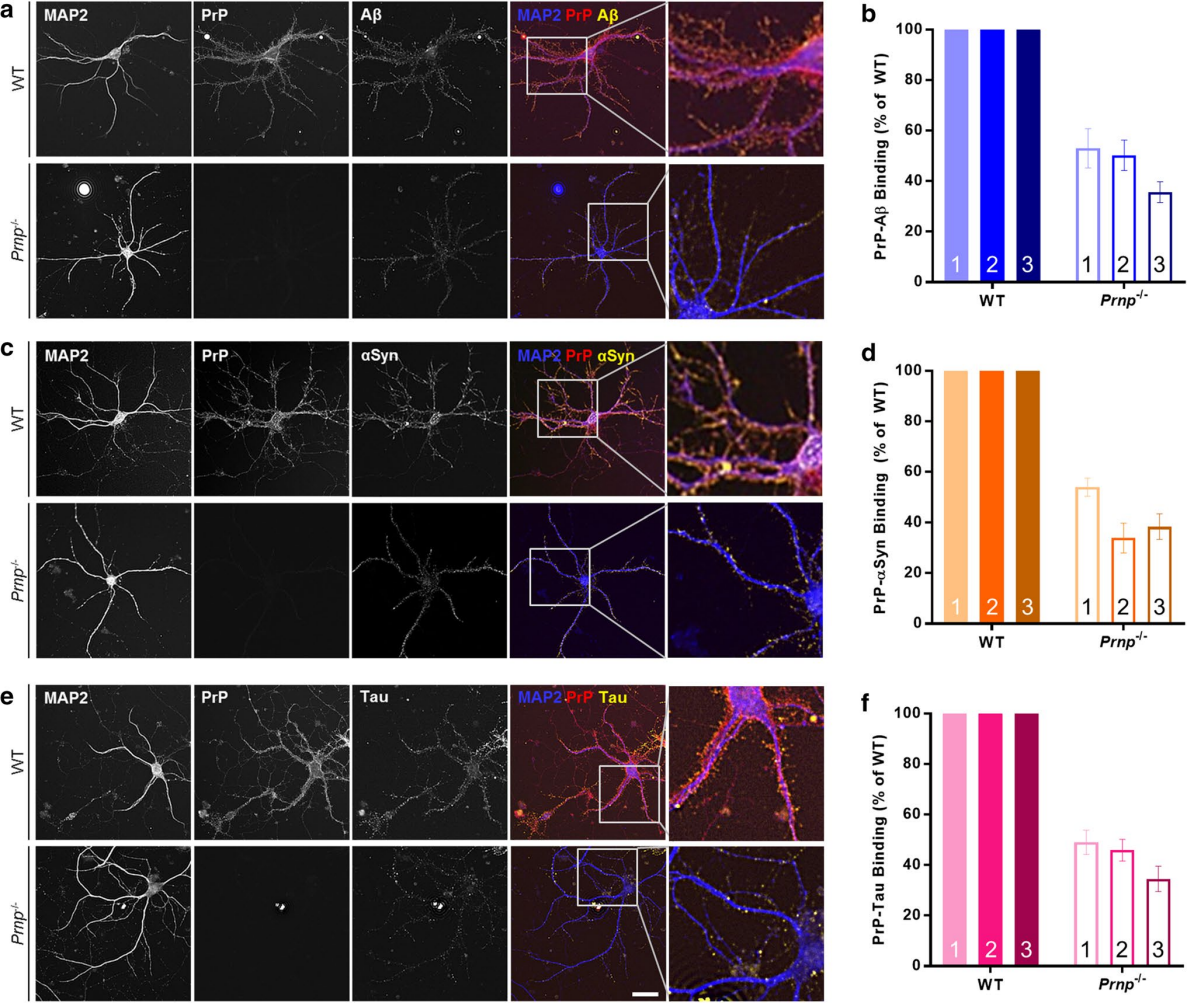
SBAs, SAAs and STAs bind to primary neurons in a PrP-dependent manner. **a**, **c** and **e** Soluble protein aggregates were added to wild-type (WT) and PrP-null (*Prnp*^−/−^) MPNs and binding assessed using serial-permeabilized immunocytochemistry. Representative images for SBAs (**a**), SAAs (**c**) and STAs (**e**) are shown. Staining for MAP2, PrP and bound proteins are shown in blue, red and yellow, respectively. Enlarged triple-colocalization images, indicated by boxed regions, are presented to the right of each panel. **b**, **d** and **f**, Relative binding of SBAs (**b**), SAAs (**d**) and STAs (**f**) to WT and *Prnp*^−/−^ MPNs was determined as in Fig. 3. Binding of soluble protein aggregates to *Prnp*^−/−^ MPNs is expressed as % binding to WT neurons. Scale bar in **a**, **c** and **e** = 50 µm, and data in **b**, **d** and **f** are the mean ± SD and represent three independent experiments with 45–300 images analyzed per experiment per genotype

To determine if binding of SPAs to neurons required PrP, we employed the same serial permeabilized ICC protocol as used above, this time on MPNs prepared from WT and PrP-null (*Prnp*^−/−^) mice (Supplementary Figure 3a). DIV21 MPNs were incubated with 500 nM SPAs for 2 h, washed and processed. Quantification of SBAs-IR puncta revealed a > 50% reduction in binding to *Prnp*^−/−^ MPNs relative to WT (Fig. 4a, b). Strikingly, SAAs-IR and STAs-IR puncta were similarly reduced in *Prnp*^−/−^ MPNs (Fig. 4c–e). Using on-cell Western blotting to monitor SPAs binding to MPNs, we also found dose-dependent binding of SPAs to WT MPNs and reduced binding to *Prnp*^−/−^ neurons (Supplementary Figure 3b–d). Furthermore, transfection of WT MPNs with short-hairpin RNAs (shRNA) targeting the 5′-untranslated region of *Prnp* (shPrP; Supplementary Figure 4a and b) greatly attenuated binding of SBAs (Supplementary Figure 4c), SAAs (Supplementary Figure 4d) and STAs (Supplementary Figure 4e) to the surfaces of neurons.

#### PrP is required for SPA-mediated neuritotoxicity and synaptotoxicity

**Fig. 5:**
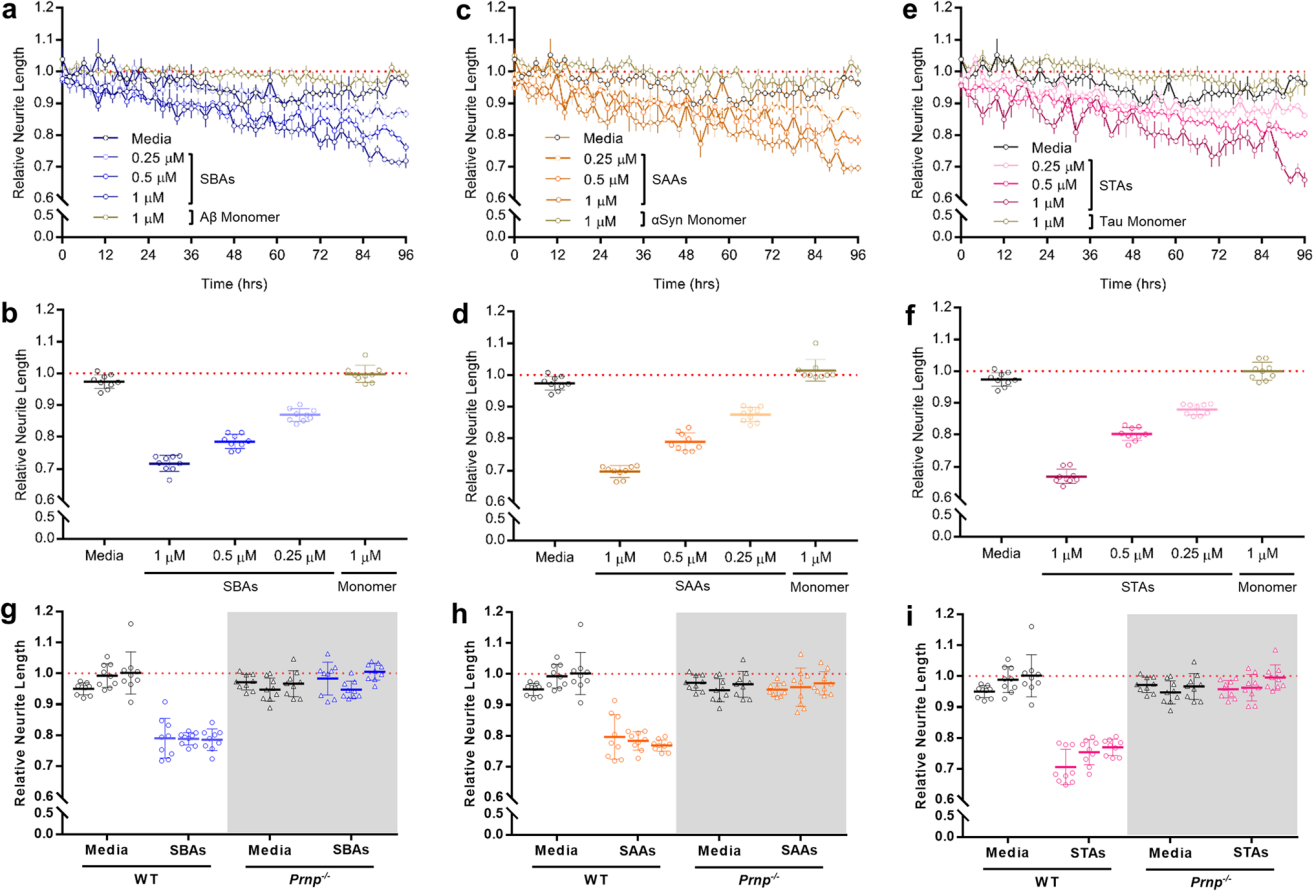
SBAs, SAAs and STAs are toxic to primary neurons in a manner requiring PrP. **a**, **c** and **e**, WT MPNs were incubated without (Media), with monomers (1 μM), or with SBAs (**a**), SAAs (**c**) and STAs (**e**) and neurite length measured using live-cell imaging. Each well was imaged for 6 h prior to the addition of samples, and NeuroTrack-identified neurite lengths were used to normalize values obtained across 96 h after sample addition. Each point represents the mean ± SEM of four images taken from three independent wells. **b**, **d**, **f** Plots of normalized neurite length for WT PMNs incubated without (Media), with monomers (1 μM), or with SBAs (**b**), SAAs (**d**) and STAs (**f**). Data are derived from the last 6 h of the traces shown in **a**, **c** and **e**, respectively. Each point represents the mean ±  SEM of 4 images from three wells for each 2 h bin. **g**, **h**, **i** Plots of normalized neurite length for WT and PrP-null (Prnp−/−; gray shading) MPNs incubated without (Media) or with SBAs (**g**), SAAs (**h**) and STAs (**i**). Data are derived from the last 6 h of treatment. Each point represents the mean ± SEM of four images from three wells for each 2 h bin from three independent experiments

We recently developed a medium-throughput, live-cell imaging assay to monitor the effects of Aβ on neuritic integrity [41, 45]. Here, we adapted this platform to assess whether SPAs or their corresponding monomers are toxic to neurons. WT MPNs were incubated with 250, 500 and 1000 nM SBAs, SAAs, STAs, or monomers and neurite length monitored for 96 h. Even at the highest concentrations tested, Aβ, αSyn and tau monomers had no effect on neurite length. In contrast, SBAs impaired neurons in a time- and dose-dependent manner (Fig. 5a, b), and highly similar effects were observed with SAAs (Fig. 5c, d) and STAs (Fig. 5e, f). Strikingly, when tested alongside WT MPNs, *Prnp*^−/−^ cells were resistant to SBAs (Fig. 5g), SAAs (Fig. 5h) and STAs (Fig. 5i).

**Fig. 6:**
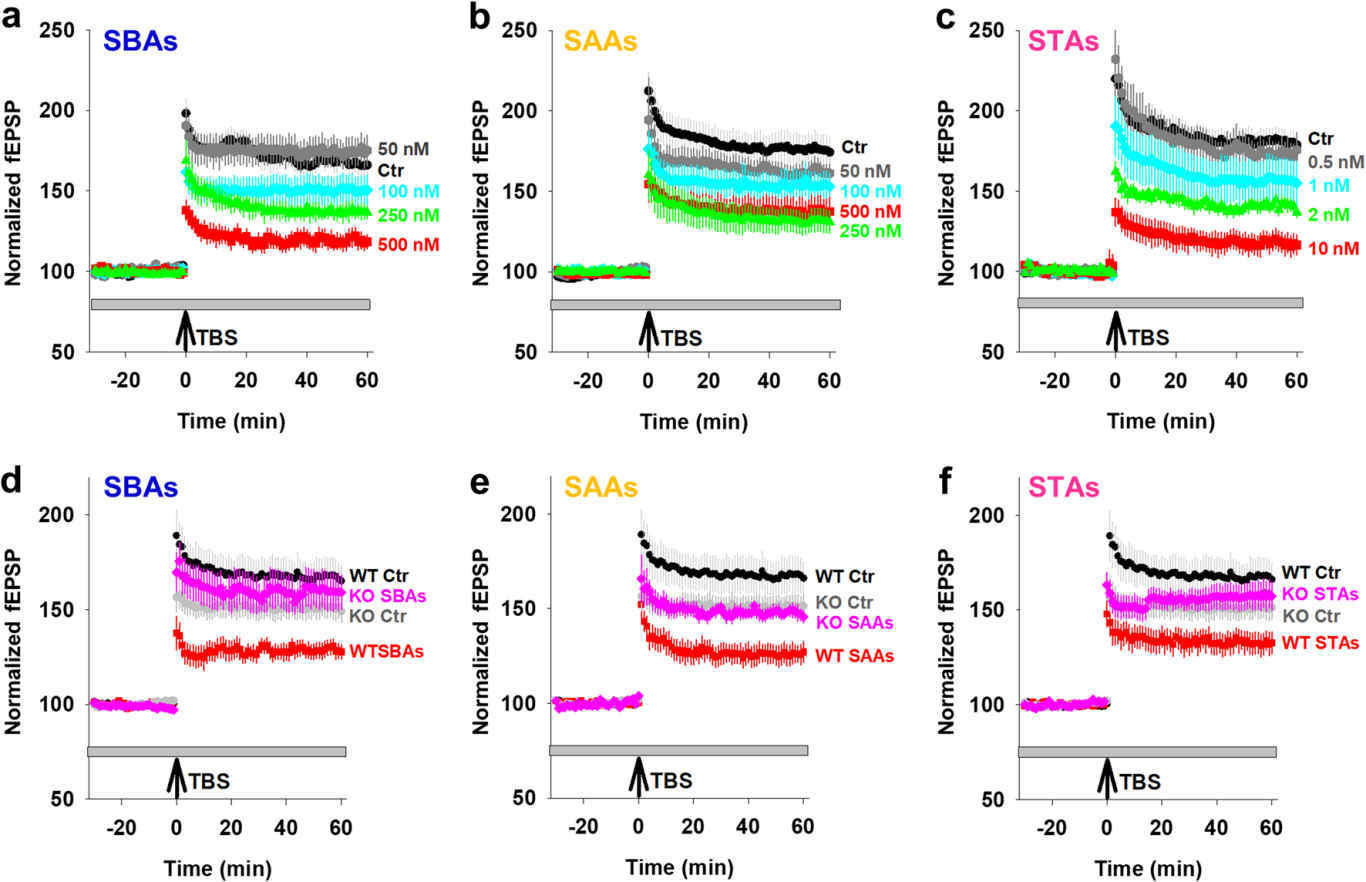
PrP is required for SPA-mediated inhibition of LTP. Field excitatory postsynaptic potential (fEPSP) were recorded simultaneously from sets of four slices using a MED64 Quad II system. **a–c** Time-course traces show that SBAs (**a**), SAAs (**b**) and STAs (**c**) dose-dependently inhibit hippocampal LTP in wild-type (WT) slices. In **a** and **b**, aCSF control (Ctr), black; 50 nM SPAs, grey; 100 nM SPAs, blue; 250 nM SPAs, green; and 500 nM SPAs, red. In **c**, aCSF control (Ctr), black; 0.5 nM SPAs, gray; 1 nM SPAs, blue; 2 nM SPAs, green; and 10 nM SPAs, red. **d–f** Time-course traces indicate that SBAs (**d**; 500 nM) and SAAs (**e**; 500 nM) and STAs (**f**; 10 nM) potently inhibit LTP in WT, but not PrP-null (*Prnp*^−/−^), slices. aCSF control on WT slices (WT Ctr), black; aCSF control on *Prnp*^−/−^ (KO Ctr) slices, gray; SPAs on WT slices, red; SPAs on *Prnp*^−/−^ slices, blue. In **a–f**, the gray horizontal bar represents the duration of SPA treatment and data represent the mean ± SD of 5–6 animals per group. Statistical analysis of results are provided in Supplementary Figure 5

Neuritic loss is a common and early facet of all neurodegenerative diseases, likely preceded by functional alterations. To assess the effects of SPAs on synaptic function, we measured long-term potentiation (LTP) in the Schaffer collaterals of mouse hippocampal slices treated with SPAs (Fig. 6a–c and Supplementary Figure 5a–c). We found that SBAs, SAAs and STAs inhibited TBS-induced LTP in a dose-dependent fashion, with SBAs and SAAs causing a significant inhibition at 250 nM. Unlike SAAs and SBAs, when STAs were tested at concentrations greater than 50 nM they completely blocked LTP. Hence, we explored lower concentrations and found that STAs caused a robust block of LTP at 2 nM. Critically, when *Prnp*^−/−^ slices were exposed to SPAs at concentrations which blocked LTP in WT slices there was no diminution of LTP. Thus, the SPA-mediated impairment of synaptic plasticity requires expression of PrP (Fig. 6d–f and Supplementary Figure 5d–f).

We previously reported that neurotoxic Aβ species isolated from AD brain impair LTP by binding directly to the presynapse [90]. Since PrP is enriched in synaptic compartments [40], we monitored colocalization of PrP and Aβ, αSyn or tau in tissue sections from three patients each who died with AD, dementia with Lewy body (DLB) and frontotemporal dementia (FTD) (Supplementary Table 1). Using high-resolution array tomography we found that presynaptic, synaptophysin-positive PrP colocalized with all three pathological proteins in end-stage disease brain (Supplementary Figure 6a and b). These human data, together with our in vitro cell-binding data (Figs. 3, 4 and Supplementary Figures 3 and 4), provide strong evidence of direct binding of SPAs with PrP.

#### An all-human experimental paradigm demonstrates that SPA-mediated toxicity requires PrP^C^

**Fig. 7:**
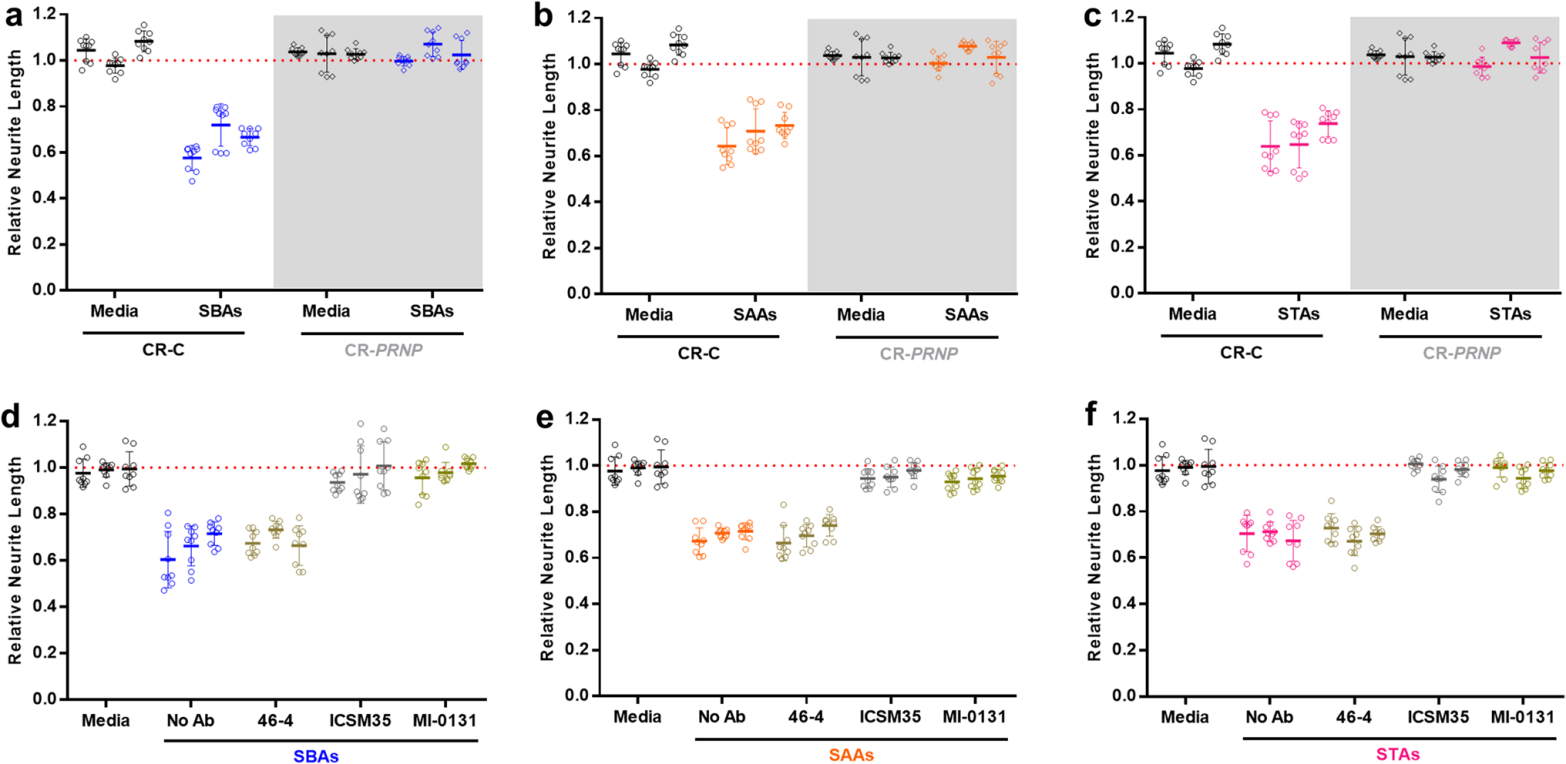
SBAs, SAAs and STAs are toxic to induced neurons in a manner requiring PrP. **a–c** iPSC-derived human neurons (iNs) expressing (CR-C) or lacking PrP (CR-*PRNP;* gray shading) were incubated without (Media) or with SBAs (**a**), SAAs (**b**) and STAs (**c**) and neurite length measured using live-cell imaging. The CR-*PRNP* line was generated using CRISPR editing (Supplementary Figure 6). Data were collected and analyzed as in Fig. 5 and represent the mean ± SEM of four images from three wells for each 2 h bin from three independent experiments. **d–f** To determine whether immunotargeting PrP could protect against neurotoxicity induced by soluble protein aggregates, iNs were treated with or without anti-PrP mAbs to *Site I* (MI-0131) or *II* (ICSM35) before the addition of SBAs (**d**), SAAs (**e**) and STAs (**f**) and neurite length measured using live-cell imaging. Data were collected and analyzed as in Fig. 5 and represent the mean ± SEM of four images from three wells for each 2 h bin from three independent experiments

To further examine the disease relevance of our experiments we utilized human iPSC-derived neurons (iNs) [96], employing CRISPR-edited isogenic lines expressing or lacking *PRNP*. CRISPR-Control (CR-C) iNs and CRISPR-*PRNP*-null (CR-*PRNP*) iNs showed a similar time-dependent elaboration of processes and were otherwise indistinguishable (Supplementary Figure 7A–C and F), except PrP null iNs lacked PrP (Supplementary Figure 7d and e). Importantly, when treated with equimolar concentrations (500 nM) of SBAs, SAAs and STAs, CR-C iNs, like WT MPNs, were susceptible to SPA-mediated toxicity. In contrast, SPAs had no measurable effect on CR-*PRNP* iN neurite length (Fig. 7a–c). Moreover, when CR-C iNs were treated with very high (10 μM) concentrations of soluble aggregates formed from bovine serum albumin, no effects on neuronal or neuritic health were observed (Supplementary Figure 8a and b).

Next, we assessed whether the two N-terminal PrP mAbs found to prevent SPA binding in our solid-phase PrP binding assays could attenuate SPA-induced loss of neuritic integrity. 500 nM SBAs, SAAs and STAs produced robust neuritotoxicity in iNs (Fig. 7d–f), whereas iNs pretreated with 3 µg/mL MI-0131 or ICSM35 were protected from these effects (Fig. 7d–f). Collectively, these results indicate that although cell surface PrP is necessary for only half of SPAs binding, PrP is required for all of the SPAs-induced neuritotoxicity.

**Fig. 8:**
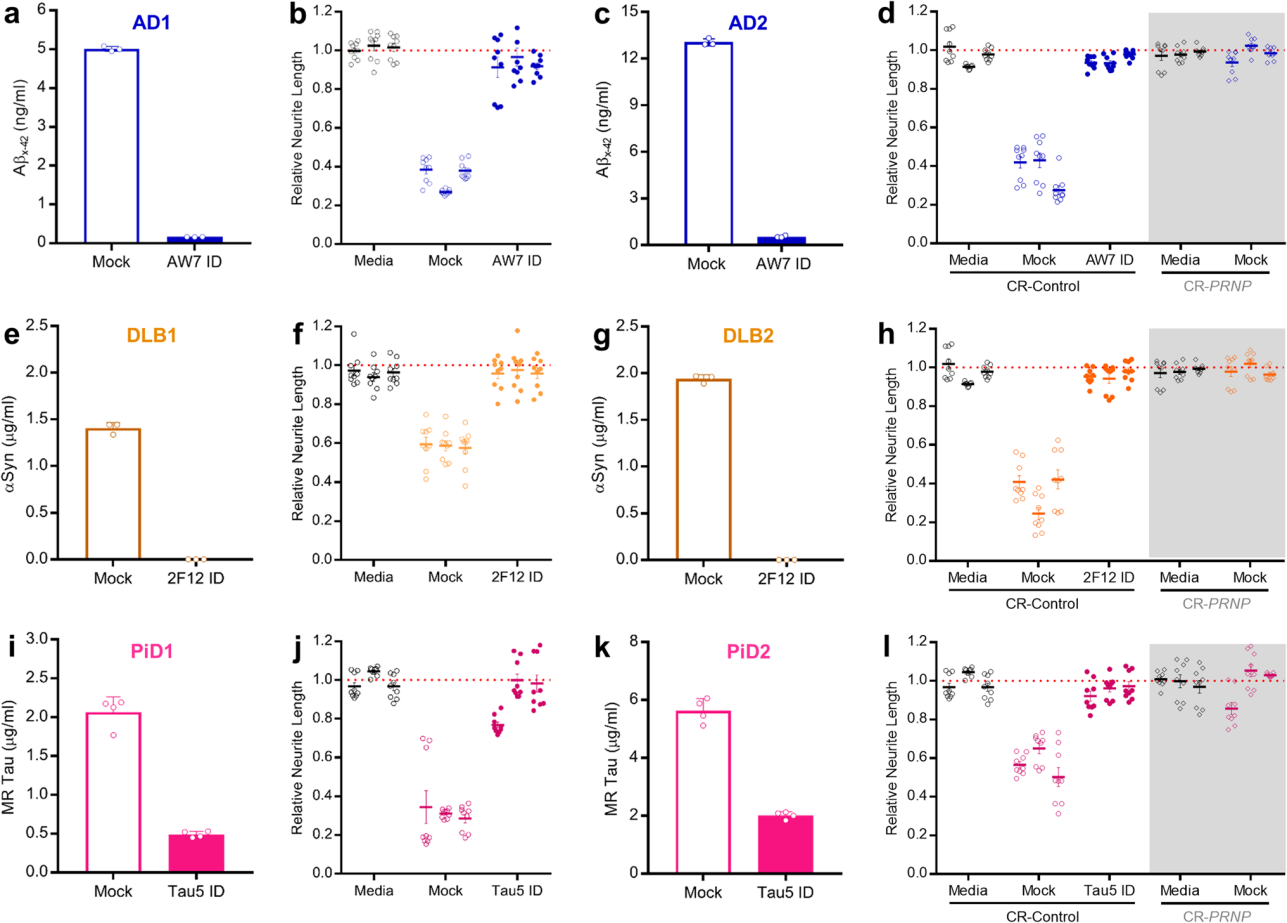
Soluble extracts from AD, DLB and PiD brains are toxic to neurons in a manner requiring PrP. **a** Aβ_*x*−42_ immunoassay quantification of Aβ present in homogenates from AD1 brain after immunodepletion with AW7 (AW7 ID) or preimmune serum (Mock). **b** iNs were incubated without (Media) or with extracts from brain AD1 ID’ed with AW7 (AW7 ID) or preimmune serum (Mock) and neurite length measured using live-cell imaging. **c** Aβ_*x*−42_ immunoassay quantification of Aβ present in homogenates from AD2 brain after immunodepletion with AW7 (AW7 ID) or preimmune serum (Mock). **d** iNs expressing (CR-Control; circles) or lacking PrP (CR-*PRNP;* diamonds, grey shading) were incubated without (Media) or with extracts from brain AD2 ID’ed with AW7 or preimmune serum (Mock) and neurite length measured using live-cell imaging. **e** ELISA quantification of αSyn present in homogenates from DLB1 brain after ID with 2F12 (2F12 ID) or isotype control (Mock). **f** iNs were incubated without (Media) or with extracts from brain DLB1 ID’ed with 2F12 (2F12 ID) or isotype control (Mock) and neurite length measured using live-cell imaging. **g** ELISA quantification of αSyn present in homogenates from DLB2 brain after ID with 2F12 (2F12 ID) or isotype control (Mock). **h** CR-Control and CR-*PRNP* iNs were incubated without (Media) or with extracts from brain DLB2 ID’ed with 2F12 (2F12 ID) or isotype control (Mock) and neurite length measured using live-cell imaging. **i** ELISA quantification of tau present in homogenates from PiD1 brain after ID with Tau5 (Tau5 ID) or isotype control (Mock). **j** iNs were incubated without (Media) or with extracts from brain PiD1 ID’ed with Tau5 (Tau5 ID) or isotype control (Mock) and neurite length measured using live-cell imaging. **k** ELISA quantification of tau present in homogenates from PiD2 brain after ID with Tau5 (Tau5 ID) or isotype control (Mock). **l**, CR-Control and CR-*PRNP* iNs were incubated without (Media) or with extracts from brain PiD2 ID’ed with Tau5 (Tau5 ID) or isotype control (Mock) and neurite length measured using live-cell imaging. In **a**, **c**, **e**, **g**, **i** and **k**, data represent the mean ± SD of three technical replicates and are representative of at least two independent experiments. In **b**, **d**, **f**, **h**, **j** and **l**, data were collected and analyzed as before and represent the mean ± SEM of four images from three wells for the last 6 h of three independent experiments

In previous studies, we found that aqueous extracts from AD brains impair memory consolidation, long-term potentiation (LTP), synaptic and neuritic structures and that these effects can be prevented by immunodepletion (ID) of Aβ [32, 41, 80]. In one case, we found that a single AD brain extract blocked LTP in a tau-dependent manner [67]. However, this extract was the exception rather than the rule. Here, we investigated the activity of soluble proteins present in aqueous extracts prepared from the brains of seven clinically and neuropathologically distinct humans: an individual who died cognitively intact and free of neurodegeneration and two patients each who died with end-stage AD, DLB and PiD (Supplementary Table 1). The procedure used to generate extracts was identical, but disease-specific brain regions were used to reflect the well-established selective vulnerability associated with AD, DLB and PiD. The AD brain extracts were ID’ed of Aβ using the anti-Aβ antiserum AW7, whereas the DLB and PiD extracts were ID’ed of αSyn and tau using mAbs 2F12 and Tau5 (Supplementary Table 2), respectively (Supplementary Figure 9). Three rounds of AW7-ID effectively removed 96% (AD1) and 97% (AD2) of the detectable Aβ as determined by x-42 MSD immunoassay (Fig. 8a, c) and Western blot (Supplementary Figure 9b; AD2). Importantly, AW7 ID did not markedly alter ELISA-measured tau levels, e.g. in AD2 tau measured 3.95 ± 0.62 μg/mL in the mock ID sample and 2.87 ± 0.21 μg/mL in the AW7 ID’d sample). Due to the higher abundance of αSyn compared with Aβ or tau, a column-based batch IP method was employed (Supplementary Figure 9c) and this removed all ELISA (Fig. 8e, g) and Western blot (Supplementary Figure 9D; DLB2) detectable αSyn from the DLB extracts. Three rounds of Tau5-ID effectively removed 78% (PiD1) and 65% (PiD2) of tau by mid-region ELISA (Fig. 8i, k), approximately 50% of phosphorylated tau in both extracts by phospho-Thr181 ELISA (PiD1 mock ID, 96.42 ± 1.26 ng/m; PiD1 Tau5 ID, 53.77 ± 3.36 ng/mL and PiD2 mock ID, 55.85 ± 1.47 ng/mL; PiD2 Tau5 ID, 21.84 ± 0.41 ng/mL) and near complete loss of the ~ 49–55 kDa bands in the Tau5 ID fraction by Western blot (Supplementary Figure 9e; PiD2).

Thereafter, we used our iN live-cell imaging paradigm to test the effects of these extracts. When used at a final dilution of 1:8, each of the extracts from diseased brains induced neuritotoxicity (Fig. 8b, d, f, h, j, l) while an extract from a subject who died free of neurodegeneration did not (Supplementary Figure 8d). ID of Aβ from the AD brain extracts rescued neuritotoxicity (Fig. 8b, d), as did ID of αSyn from the DLB brain extracts (Fig. 8f, h), and ID of tau from the PiD brain extracts (Fig. 8j, l). To determine if PrP was involved in the observed toxicity, CR-*PRNP* iNs were exposed to mock-ID extracts. Strikingly, in three separate experiments, knock-out of PrP protected iNs from neuritotoxicity mediated by the extracts of AD, DLB and PiD brains (Fig. 8d, h, l, shaded regions). These unambiguous findings imply a central role for PrP in SPA-mediated toxicity.

## Discussion

Prion diseases typically involve a long, silent period of PrP aggregation and propagation, and an explosive symptomatic phase which results from acute neurotoxicity [1, 54, 75]. While the mechanism(s) causing neuronal loss are not yet resolved it is clear that membrane-bound PrP^C^ is required [12, 48, 58, 65] and binding of misfolded PrP to PrP^C^ is a critical step [11]. β-sheet rich assemblies formed from design peptides, yeast prion and Aβ, can also bind to PrP^C^ and cause neuronal compromise [74]. This raises the intriguing possibility that PrP^C^ may contribute to other proteinopathies by facilitating a neurotoxic signaling cascade [7, 74].

Mindful of the inconsistencies which have plagued the study of protein aggregation and confounded assessment of bioactivity [8, 79, 89], we developed a standardized protocol to prepare soluble aggregates and accurately determine their concentration. For this we exploited our highly reproducible system [38] which has been used to elucidate the microscopic events underlying Aβ aggregation [2, 21]. Central to our approach was the use of rigorously controlled conditions which yield highly homogenous end-stage protein aggregates, their solubilization using a standardized sonication procedure, and careful quantitation using validated ELISAs. This not only allowed us to accurately compare the binding and toxicity of monomers, soluble aggregates and fibrils formed by a single protein, but also those formed by different proteins.

Using a sensitive ELISA-like assay and binding to PrP-expressing and -deficient cells, we demonstrate that soluble aggregates of Aβ, αSyn and tau readily bind to PrP, whereas corresponding monomers and fibrils show little affinity. Across multiple experiments, SBAs and SAAs showed similar binding (EC_50_ ~ 30 nM) to full-length PrP, whereas STAs consistently showed slightly tighter binding (EC_50_ ~ 10 nM). It is interesting to speculate that this stronger binding might be related to the more potent block of LTP exerted by STAs compared with SBAs and SAAs. Heparin is known to bind PrP [17] and the heparin used to induce tau aggregation could potentially explain the enhanced binding of STAs to PrP. However, this is unlikely since a sensitive assay detected no heparin in the tau fibrils used to produce STAs. Moreover, fibrils that were formed in the presence of heparin, but not solubilized, bound only weakly to PrP. Notably, fibrils produced from recombinant monomer formed both straight and twisted filaments analogous to species found in human tauopathies. Thus, it is unlikely end-stage tau fibrils, regardless of the methods used for their generation or purification, interact with PrP.

Use of deletion constructs revealed some differences in SPA binding to PrP. SBAs exhibited appreciable binding to a construct, PrP_91–231_, which lacks binding *Site I* and retains binding *Site II*. In contrast, neither SAAs nor STAs bound PrP_91–231_ to a significant extent. Why this should be is as yet unclear, but likely derives from ultrastructural differences between SBAs, SAAs and STAs. Conversely, when applied to cortical neurons there was no discernible difference in binding or toxicity of the three different SPAs. The inability to detect a difference in binding to neurons is understandable given that approximately 50% of SPA binding is not mediated by PrP and that ICC is less quantitative than our ELISA-like assay. Co-immunoprecipitation is a common method used to probe for interactions between endogenous proteins. However, PrP^C^ is a membrane-attached protein that requires the use of strong detergents for its solubilization making it difficult to identify non-covalent interactions sensitive to detergents.

Importantly, both the plasticity disruption and neuritotoxicity induced by SPAs were completely absent when PrP was ablated. Though probing the mechanism by which PrP mediates these effects is beyond the scope of the current study, it is interesting to note that the PrP is known to complex with metabotropic glutamate receptors (mGluRs) and that mGluRs have been linked to PrP-mediated disruption of LTP [43, 84] and changes in neuritic architecture [9]. Indeed, our data indicate that all three SPAs caused LTP and structural impairments, albeit to different extents. For example, we observed that, relative to other SPAs, STAs bind tighter to PrP and impair LTP at much lower concentrations. However, equimolar concentrations of SPAs cause nearly equal neuritotoxicities. One possible explanation for this is the differences between acute slice physiology and in vitro cell-structural models and the fact that physiological changes are thought to precede structural alterations. Regardless, the lack of effect of SPAs on PrP-null hippocampal slices and dissociated neurons indicates that the effect of SPAs on WT cells is not a consequence of mechanical disruption since membranes should be equally susceptible to pore forming assemblies irrespective of PrP expression. This does not exclude the possibility that other forms of Aβ, αSyn and tau can induce toxicity by PrP-independent mechanisms. Nonetheless, the fact that extracts from diseased brains cause neuritoxicity in a PrP-dependent manner strongly suggests that the bioactive forms of SPAs present in human brain share some similarity with SPAs generated in vitro. Moreover, our finding that PrP antibodies prevent AD, DLB and PiD brain-induced toxicity argues against the lack of toxicity in PrP null neurons being due to unknown protective effects of constitutive PrP ablation. Similarly, amelioration of toxicity by immunodepletion with antibodies specific for Aβ, αSyn and tau demonstrates the involvement of these proteins. Furthermore, the lack of effect of control brain extracts rich in αSyn and tau indicates that the toxicity seen with DLB and PiD brain extracts is imparted by disease-specific, presumably aggregated, forms of these proteins.

While numerous studies have demonstrated that soluble aggregates of Aβ can bind to PrP and induce a range of toxicities, much less is known about whether αSyn and tau interact with PrP. A paper published during the submission of our manuscript claimed to have been able to co-IP PrP, APP, Aβ and phospho-tau from transgenic mouse and human brain [34]. Here, we show direct binding of tau to PrP in vitro, that this interaction is aggregation-dependent and necessary for SPA-mediated disruption of LTP and neuritotoxicity. Moreover, we provide complementary evidence of binding using mouse primary neurons, iPSC-derived human neurons, and array tomography of diseased brains, that together substantiate the in vivo relevance of the SPA-PrP interaction. Two previous studies investigated binding of αSyn to PrP [29, 86], both of which used what they referred to as oligomers. Intriguingly, the preparation used in the study that reported toxicity mediated by αSyn that was not dependent on PrP^C^ appeared as imperfect spheres with average diameters less than 20 nm [86]. Comparable structures are formed by Aβ and these too bind only weakly to PrP, and like αSyn pseudo-spheres mediate toxicity independent of PrP^C^ [66]. In contrast, short Aβ fibrils and protofibrils, similar to our SAAs, SBAs and STAs, bind to PrP and require PrP^C^ to induce toxicity [66]. Collectively, these results suggest that protofibrils are the principle species which bind to PrP and mediate toxicity via PrP^C^. A prior study reported that αSyn oligomers induced PrP-dependent toxicity in a number of different paradigms, but the authors did not provide detailed analysis of the active αSyn preparation and used antibodies (without validating binding activity) to infer potential PrP binding sites. Here, we employed well-validated antibodies and deletion constructs to systematically demonstrate that SAAs, but not αSyn monomer or fibrils, bind to PrP and that binding was mediated via binding *Sites I* and *II*. Moreover, SPAs which bind PrP caused neuritotoxicity and impairment of LTP, while monomers and fibrils did not. Thus, our results clearly link PrP binding with toxicity.

When breaking ground in a new area no single study can answer all the questions raised by novel findings, nor apply the full gambit of approaches in the armamentarium of modern biomedicine. So it is with our study. While we have unambiguously demonstrated that both recombinant and human brain-derived forms of Aβ, tau and αSyn can bind to PrP and induce functional and structural deficits, this important beachhead requires strengthening and expansion. It will fall to future studies to purify soluble protein aggregates from diseased brain and to test their toxic activities. In this regard, our recent observation that recombinant PrP N1 can protect against the toxicity of brain-derived Aβ [62] suggests that PrP derivatives such as N1 may serve as affinity reagents to enable the isolation and molecular identification of SPAs. Similarly, in vivo studies will be required to further investigate the translational nature of our work. Namely, experiments in tau and αSyn transgenic mice should test the value of targeting PrP, by genetic knock-out, or knock-down, or use of anti-PrP antibodies. In this era when antisense oligonucleotide and RNAi technologies are being successfully applied for treatment of what were previously thought to be incurable diseases [55], experiments in mice could be a prelude to knocking-down PrP^C^ expression in humans [65]. Such an approach is supported by the fact that loss of one PrP^C^ allele is well tolerated in humans [63]. Thus, given our work suggests that targeting PrP^C^ could be of benefit in several neurodegenerative diseases we recommend the exploration of PrP^C^ as a therapy which could be particularly useful in individuals with evidence of mixed pathologies.

## Electronic supplementary material

Below is the link to the electronic supplementary material.

Supplementary file1 (PDF 1441 kb)

## Abbreviations

Aβ: Amyloid β-protein
AD: Alzheimer's disease
ADDLs: Aβ-derived diffusible ligands
aSyn: α-Synuclein
CBD: Corticobasal degeneration
CR-C: CRISPR-control
CRISPR: Clustered regularly interspaced short palindromic repeats
CR-PRNP: CRISPR-*PRNP* null
D*IV*: Days in vitro
DLB: Dementia with lewy body
ICC: Immunocytochemistry
ID: Immunodepletion
iN: IPSC-derived induced neurons
iPSC: Induced pluripotent stem cell
IR: Immunoreactive
LTP: Long-term potentiation
mAb: Monoclonal antibody
mGluR: Metabotropic glutamate receptors
MPN: Mouse primary neurons
PD: Parkinson's disease
PiD: Pick's disease
*PRNP*: Prion protein gene
PrP: Prion protein
PrP^C^: Cellular prion protein
PrP^Sc^: Prion protein-scrapie
PSP: Progressive supranuclear Palsy
SAAs: Soluble α-synuclein aggregates
SBA: Soluble Aβ aggregates
SEC: Size exclusion chromatography
SPA: Soluble protein aggregates
STAs: Soluble Tau aggregates
TBS: Theta-burst stimulation
ThT: Thioflavin T
WT: Wild type
