# Supplementary material for: PrP is a central player in toxicity mediated by soluble aggregates of neurodegeneration-causing proteins
Source: Acta Neuropathol. 2019 Dec 18;139(3):503–26. doi: 10.1007/s00401-019-02114-9 (PMC7035229; doi:10.1007/s00401-019-02114-9)
Supplement: Supplementary file 1 — Supplementary file1 (PDF 1441 kb) [file 401_2019_2114_MOESM1_ESM.pdf]

PrP is a central player in toxicity mediated by soluble aggregates of neurodegeneration-causing proteins

Grant T. Corbett[1], Zemin Wang[1], Wei Hong[1], Marti Colom-Cadena[2], Jamie Rose[2], Meichen Liao[1], Adhana Asfaw[1], Tia C. Hall[1], Lai Ding[3], Alexandra DeSousa[1], Matthew P. Frosch[4,] John Collinge[5], David A. Harris[6], Michael S. Perkinton[7], Tara L. Spires-Jones[2], Tracy L. Young-Pearse[1], Andrew Billinton[7], and Dominic M. Walsh[1]*

[1]Laboratory for Neurodegenerative Research, Ann Romney Center for Neurologic Diseases, Brigham & Women's Hospital and Harvard Medical School, Boston, MA 02115, USA
[2]Centre for Discovery Brain Sciences and UK Dementia Research Institute, University of Edinburgh, Edinburgh, UK EH89JZ
[3]Program for Interdisciplinary Neuroscience, Brigham & Women's Hospital, Boston, MA 02115, USA
[4]Massachusetts General Institute for Neurodegenerative Disease, Massachusetts General Hospital and Harvard Medical School, Charlestown, MA 02129, USA
[5]MRC Prion Unit at UCL, UCL Institute of Prion Diseases, National Hospital for Neurology and Neurosurgery, Queen Square, London WC1N 3BG, UK
[6]Department of Biochemistry, Boston University School of Medicine, Boston, MA 02118, USA
[7]Neuroscience, IMED Biotechnology Unit, AstraZeneca, Cambridge, CB21 6GH, UK

***To whom correspondence should be addressed:**
Dominic M. Walsh
Laboratory for Neurodegenerative Research
Ann Romney Center for Neurologic Diseases
Brigham & Women's Hospital
Hale Building for Transformative Medicine (1000-2O)
60 Fenwood Road
Boston, MA 02115
Tel: 1-617-525-5059, Fax: 1-617-525-5252
Email: dwalsh3@bwh.harvard.edu

Supplementary Materials

Supplementary Figure 1. PrP constructs contain significant α-helical structure and are recognized by anti-PrP mAbs

Supplementary Figure 2. Aβ, α-synuclein and tau monomers do not bind to primary neurons

Supplementary Figure 3. Soluble protein aggregates bind to primary neurons in a dose and PrP-dependent manner

Supplementary Figure 4. shRNA targeting of PrP attenuates binding of soluble protein aggregates to neurons

Supplementary Figure 5. SPAs impair LTP in a dose- and PrP-dependent manner

Supplementary Figure 6. Aβ, αSyn and tau colocalize with PrP in human brain tissue.

Supplementary Figure 7. Preparation of iNs and characterization of CRISPR control and *PRNP*-depleted iNs

Supplementary Figure 8. Neither soluble aggregates formed from BSA nor non-disease control brain extracts cause neuritotoxicity

Supplementary Figure 9. Preparation of soluble brain extracts and immunodepletion of Aβ, α-synuclein and tau

Supplementary Table 1. Demographic details of the cases used in toxicity and array tomography studies

Supplementary Table 2. Primary antibodies and their antigens, dilutions and sources

Supplemental Figures

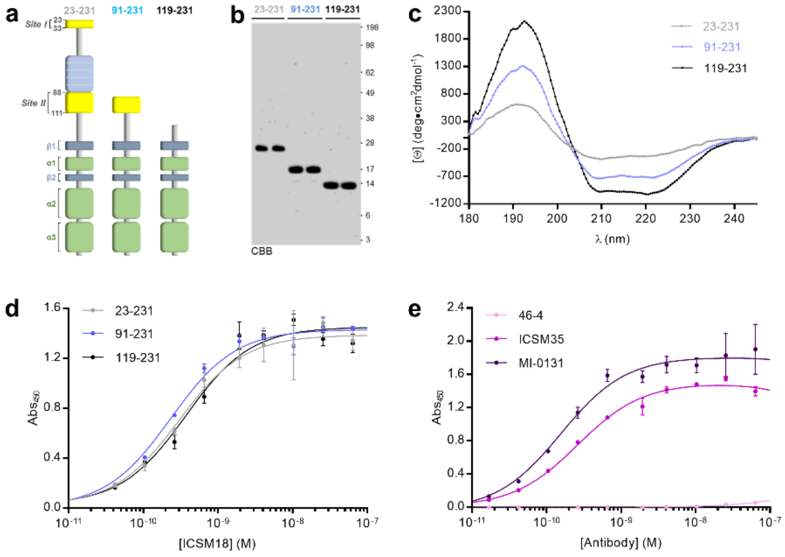

Supplementary Figure 1. PrP constructs contain significant α-helical structure and are recognized by anti-PrP mAbs. **a**, Graphic depicting the domain structure of $PrP_{23\text{-}231}$ (grey), $PrP_{91\text{-}231}$ (blue) and $PrP_{119\text{-}231}$ (black). Putative Aβ binding *Sites I* (23-33) and *II* (88-111) are indicated in yellow. **b**, Coomassie (CBB)-stained SDS-PAGE of $PrP_{23\text{-}231}$ (grey), $PrP_{91\text{-}231}$ (blue) and $PrP_{119\text{-}231}$ (black), each loaded at 500 ng/well. Molecular weight markers (in kDa) are indicated on the right. **c**, Circular dichroism (CD) spectra of $PrP_{23\text{-}231}$ (grey), $PrP_{91\text{-}231}$ (blue) and $PrP_{119\text{-}231}$ (black). **d**, Binding of the anti-PrP mAb ICSM18 to $PrP_{23\text{-}231}$ (grey), $PrP_{91\text{-}231}$ (blue) and $PrP_{119\text{-}231}$ (black) coated microtiter plates. **e**, Binding of the anti-PrP mAbs ICSM35 (light blue) and MI-0131 (dark blue) to $PrP_{23\text{-}231}$. The nonspecific mAb, 46-4 (grey), was included as negative control. Data in **c-e** are the mean ± SD of three technical replicates and are representative of at least 3 independent experiments.

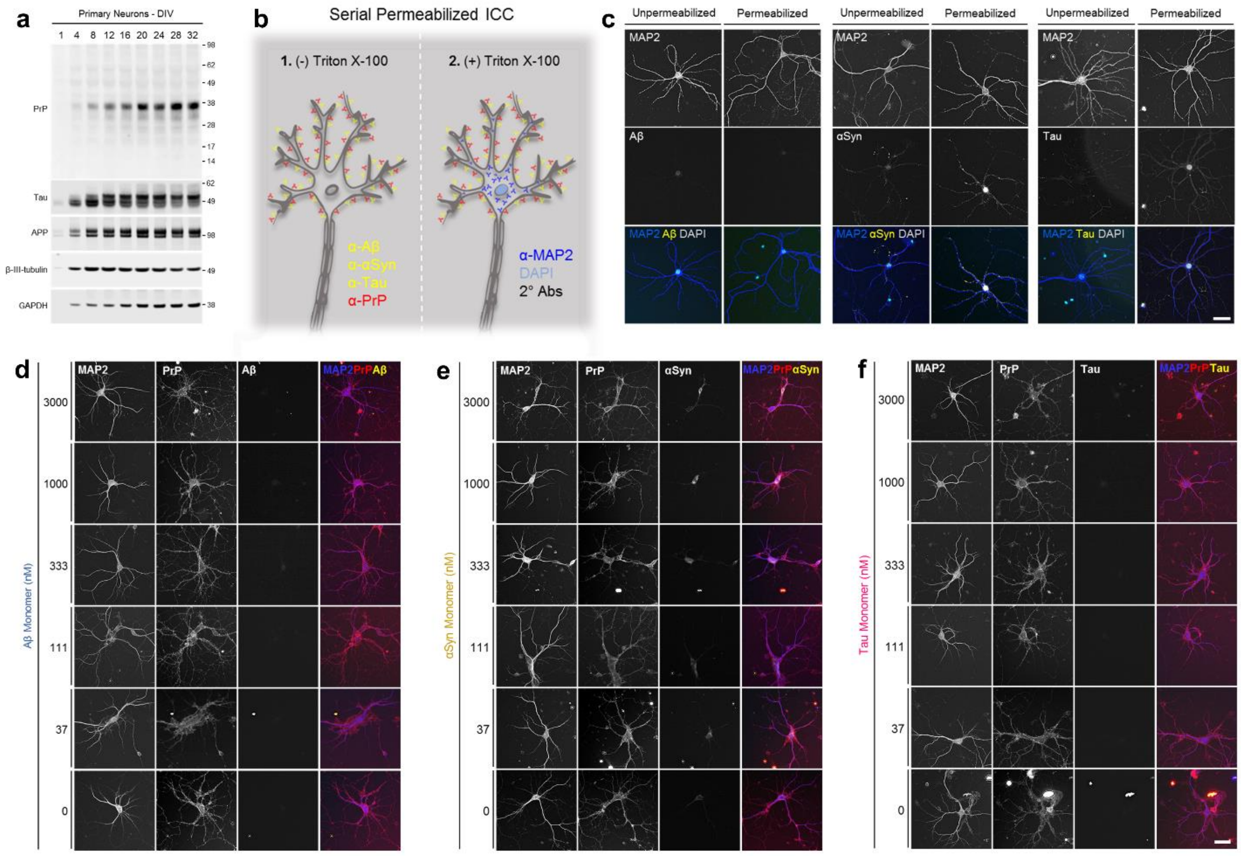

Supplementary Figure 2. Aβ, α-synuclein and tau monomers do not bind to primary neurons. **a**, Mouse primary neurons (MPNs) were seeded in 6-well plates, lysed every 4 d until day *in vitro* (DIV) 32 and analyzed by Western blotting. PrP, tau and APP were detected with ICSM18, K9JA and C1/6.1, respectively, and β-III-tubulin and GAPDH served as loading controls. Molecular weight markers (in kDa) are indicated on the right. **c**, Graphic depicting the procedure for serial permeabilized immunocytochemistry (ICC). After fixation, surface proteins are detected without permeabilization (left; red and yellow) prior to revealing intracellular epitopes (right; blue). **C**, Untreated MPNs were fixed and stained with antibodies against Aβ (3D6; left), αSyn (2F12; center), or tau (HJ8.5; right) before or after permeabilization. **d-f**, Freshly isolated monomers were added to MPNs and binding assessed using serial-permeabilized immunocytochemistry. Representative images Aβ (**d**), αSyn (**e**) and tau (**f**) are shown. Staining

for MAP2, PrP and bound proteins are shown in blue, red and yellow, respectively. Scale bar in **c** and **d-f** = 50 µm.

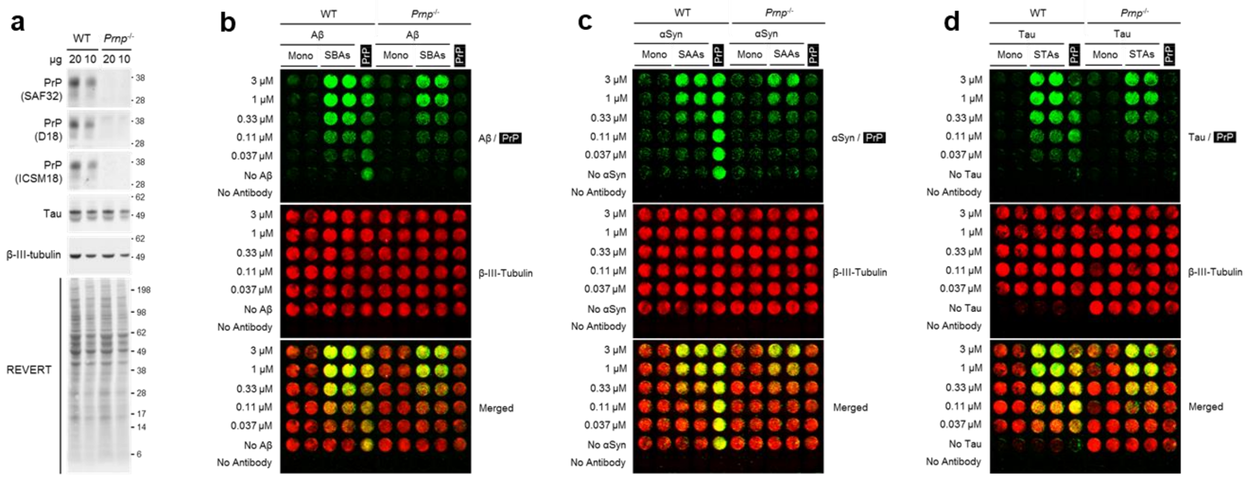

Supplementary Figure 3. Soluble protein aggregates bind to primary neurons in a dose and PrP-dependent manner. a, Western blot analysis of PrP expression in wild-type (WT) and PrP-KO (*Prnp*$^{-/-}$) mouse primary neuron (MPNs) lysates. Ten and twenty micrograms of each lysate were loaded. PrP was detected with SAF32, D18 and ICSM18 and tau (K9JA), β-III-tubulin and REVERT (total protein stain) served as loading controls. Molecular weight markers (in kDa) are indicated on the right. **b-d**, Freshly isolated monomers and soluble protein aggregates were added to WT and *Prnp*$^{-/-}$ MPNs and binding assessed using two-color, infrared on-cell western blot analysis. Representative images of Aβ (**b**), αSyn (**c**) and tau (**d**) binding are shown and Aβ, αSyn and tau were detected with 3D6, 4B12 and HJ8.5, respectively. PrP (black shading) was detected with ICSM18 and β-III-tubulin served as signal control. The images represent the plates exactly as they were scanned.

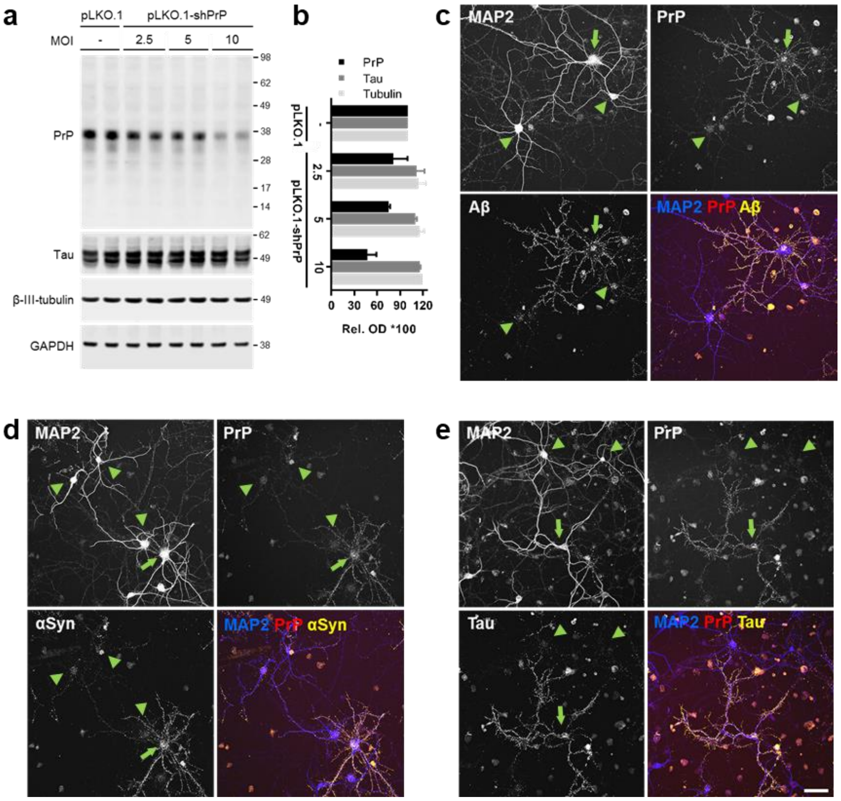

Supplementary Figure 4. shRNA targeting of PrP attenuates binding of soluble protein aggregates to neurons. a. Western blot analysis of PrP expression in WT MPNs transduced with empty vector (pLKO.1) or PrP-targeted (pLKO.1-shPrP) short hairpin RNAs at various multiplicities of infection (MOI). PrP was detected with ICSM18, and tau (K9JA), β-III-tubulin and GAPDH served as neuronal health and loading controls. Molecular weight markers (in kDa) are indicated on the right. **b**, Densitometric quantification of PrP, tau and β-III-tubulin (relative to GAPDH) in blots from A. **c-e**, Soluble protein aggregates were added to pLKO.1-shPrP-transduced MPNs and binding assessed using serial-permeabilized immunocytochemistry. Representative images SBAs (**b**), SAAs (**c**) and STAs (**d**) are shown. Staining for MAP2, PrP and bound proteins are shown in blue, red and yellow, respectively. Due to random infection of

cells, each micrograph depicts at least one transduced and one non-transduced neuron. Scale bar = 50 µm.

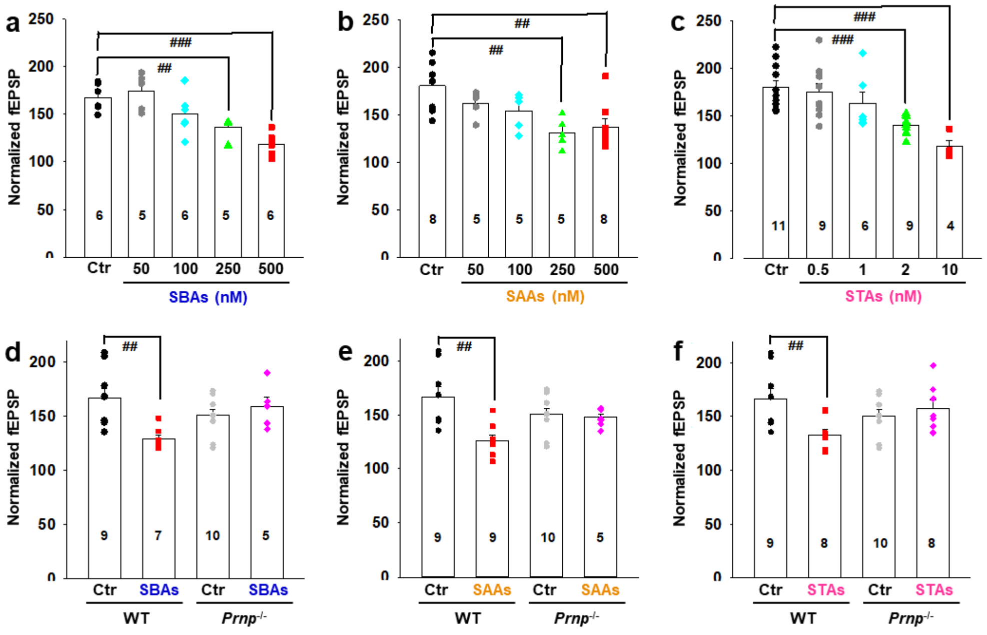

Supplementary Figure 5. SPAs impair LTP in a dose- and PrP-dependent manner. a-c, Histograms of the average field excitatory postsynaptic potentials (fEPSP) for the last 10 min of the traces shown in **Figure 6a-c** indicate that SBAs (**a**; Ctr vs 50 nM SBAs, F=5.12, p=0.54; Ctr vs 100 nM SBAs, F=4.96, p=0.14; Ctr vs 250 nM SBAs, F=5.12, p=0.003; Ctr vs 500 nM SBAs, F=4.96, p=7.87E^{-005}), SAAs (**b**; Ctr vs 50 nM SAAs, F=4.84, p=0.3; Ctr vs 100 nM SAAs, F=4.84, p=0.13; Ctr vs 250 nM SAAs, F=4.84, p=0.006; Ctr vs 500 nM SAAs, F=4.6, p=0.009) and STAs (**c;** Ctr vs 0.5 nM STAs, F=4.41, p=0.63; Ctr vs 1 nM STAs, F=4.54, p=0.22; Ctr vs 2 nM STAs, F=4.41, p=0.0002; Ctr vs 10 nM STAs, F=4.6, p=0.0003) dose-dependently inhibit hippocampal LTP. **d-f**, Histograms of the fEPSP for the last 10 min of the traces shown in **Figure 6d-f** indicate that 500 nM SBAs (**d**), SAAs (**e**) and STAs (**f**) potently inhibit LTP in wild-type (WT; Ctr vs 500 nM SBAs, F=4.75, p=0.003; Ctr vs 500 nM SAAs, F=4.49, p=0.001; Ctr vs 10 nM STAs, F=4.54, p=0.007), but not PrP-null

(*Prnp*$^{-/-}$; Ctr vs 500 nM SBAs, F=4.96, p=0.87; Ctr vs 500 nM SAAs, F=4.54, p=0.7; Ctr vs 10 nM STAs, F=4.49, p=0.44), slices. Each dot represents the average potentiation of the last 10 min of recording from each individual slice, the number of slices analyzed per group are indicated within the bars and data represent the mean ± SD.

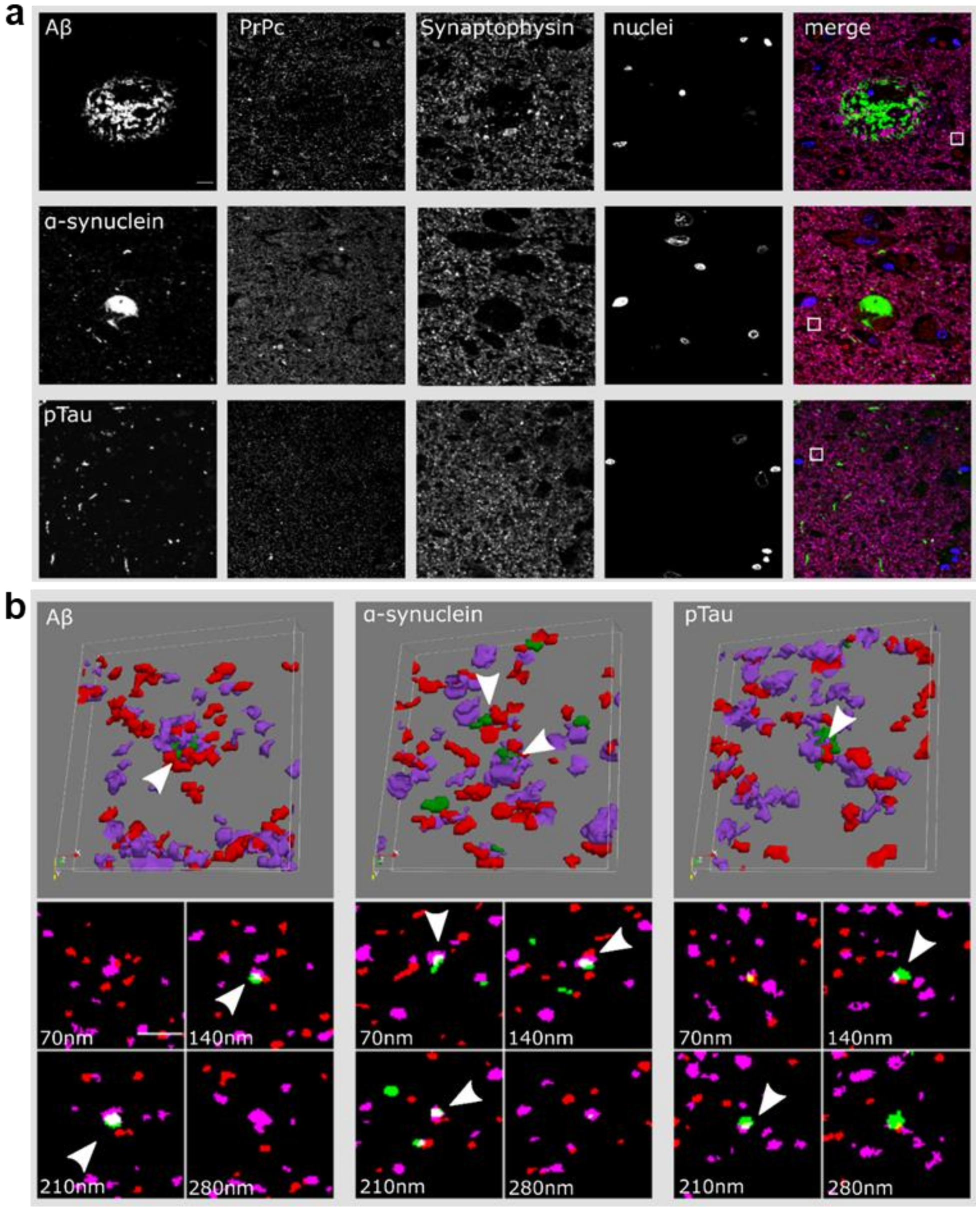

Supplementary Figure 6. Aβ, αSyn and tau colocalize with PrP in human brain tissue. Array tomography imaging shows synaptic localization of PrP at synapses together with Aβ, αSyn or tau in human postmortem brain. **a**, raw images of average intensity projections of 10 consecutive, 70 nm thick sections are shown. The top row depicts a representative AD case with Aβ deposition, the second row a DLB case with αSyn pathology, and the third row a PiD case

with abundant pTau positive neuropil threads. In the last column is shown the overlay image of the pathological protein of the first column (green), PrP (red), synaptophysin (magenta) and nuclei (blue). **b**, processed and segmented images of the insets outlined of the last column of **a**. The first row shows 3D reconstructions of 10 serial sections (6.5 x 6.5 x 0.7 μm), with white arrowheads indicating points of overlap. The second row shows 4 consecutive 70 nm thick images with overlap between PrP, synaptophysin and a pathological protein is indicated by the arrowheads. Scale bars: A, 10μm: B, 2μm.

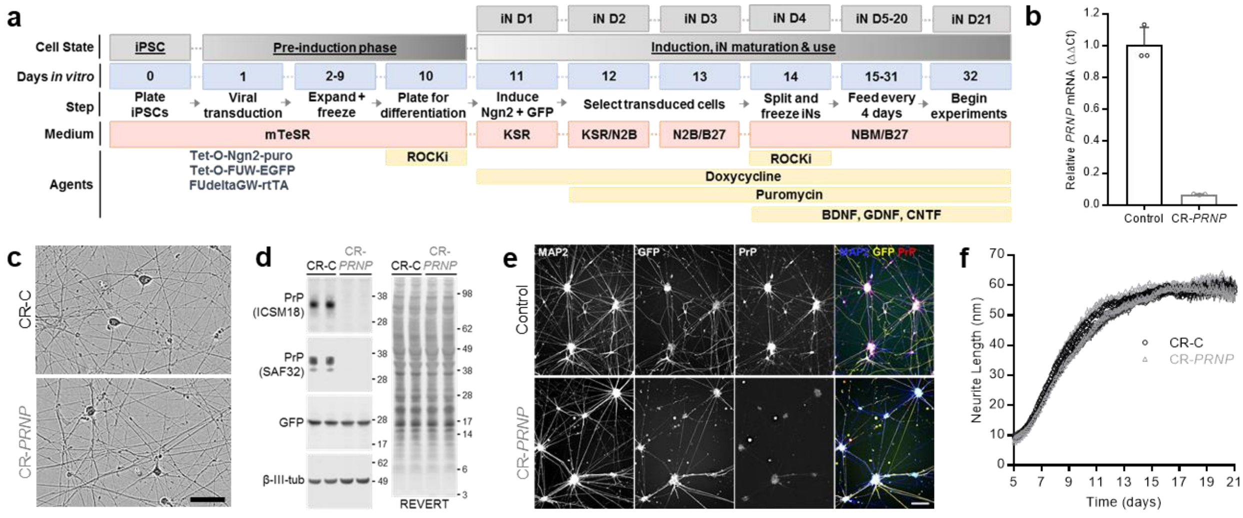

Supplementary Figure 7. Preparation of iNs and characterization of CRISPR control and *PRNP*-depleted iNs. **a**, Schematic showing the protocol used to differentiate induced pluripotent stem cells (iPSCs) into induced neurons (iNs). **b**, Relative *PRNP* mRNA expression in CRISPR control (CR-C) and PrP targeted (CR-*PRNP*) iNs. **c**, Representative phase micrographs of live-imaged CR-C (top) and CR-*PRNP* (bottom) iNs at D21. Scale bar = 25 µm. **d**, WB analysis of PrP expression in CR-C (top) and CR-*PRNP* (bottom) iN lysates at D21. PrP was detected with ICSM18 and SAF32, and GFP, β-III-tubulin and REVERT served as loading controls. Molecular weight markers (in kDa) are indicated on the right. **e**, On iN D21, CR-C (top) and CR-*PRNP* (bottom) iNs were fixed and stained for MAP2 (blue), GFP (yellow) and PrP (red). Scale bar = 100 µm. **f**, CR-C and CR-*PRNP* iNs were plated (iN D4), transferred to an IncuCyte system, and neurite development monitored every 2 h until iN D21. Each point represents the mean ± SEM of 4 images taken from 6 separate wells.

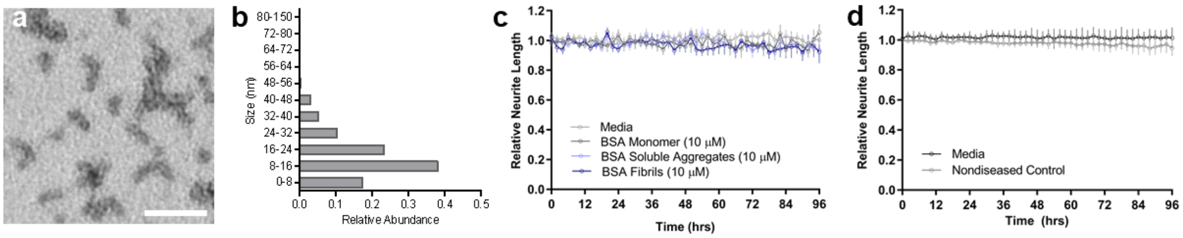

Supplementary Figure 8. Neither soluble aggregates formed from BSA nor non-disease control brain extracts cause neuritotoxicity. a, Representative negative-stain EM micrographs of sonicated, soluble BSA aggregates. **b**, Size distribution of soluble BSA aggregates as determined by negative-stain EM. **c**, CR-Control iNs were incubated without (Media) or with 10 μM BSA monomer, soluble aggregates or fibrils and neurite length measured using live-cell imaging. **d**, CR-Control iNs were incubated without (Media) or with extracts from a non-diseased control subject and neurite length measured using live-cell imaging. In **c** and **d**, data were collected and analyzed as in Figures 5, 7, and 8.

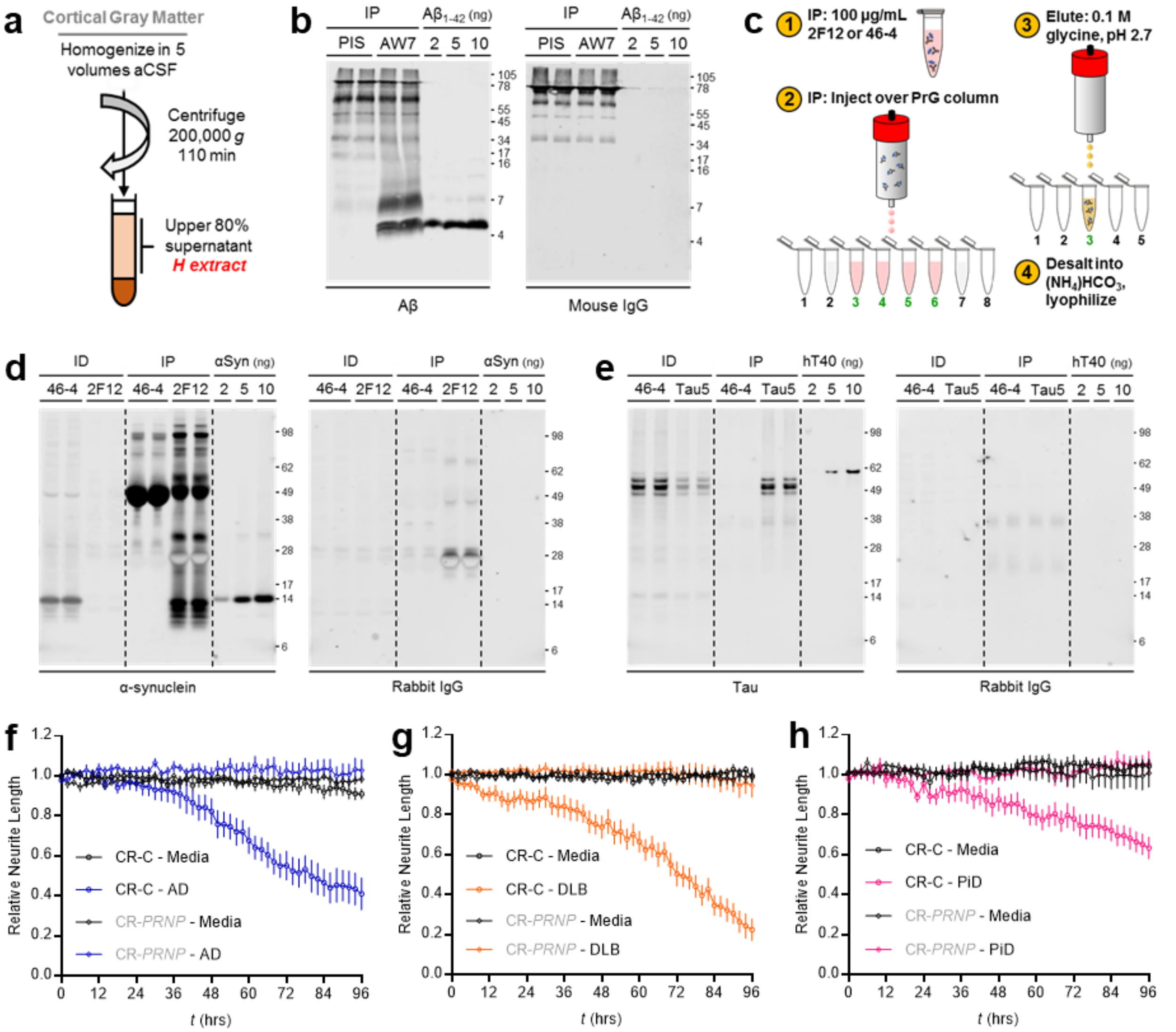

Supplementary Figure 9. Preparation of soluble brain extracts and immunodepletion of Aβ, α-synuclein and tau. **a**, Graphic depicting the procedure for preparing aqueous extracts from *post mortem* brain tissue. **b**, Western blot (WB) analysis of AW7 and preimmune serum (PIS) immunoprecipitates (IP) recovered from the AD brain extract. Synthetic Aβ$_{1-42}$ was loaded as positive control, and proteins detected with 2G3 and 21F12 (left). Identical blots were developed with 46-4 (right). **c**, Graphic depicting the protocol for immunodepletion of αSyn from aqueous brain homogenates. (1) 1 ml extract was incubated with 100 µg 2F12 or 46-4

overnight, (2) injected over a 1 ml Protein G column (PrG) and the resulting elution peak collected (fractions 3-6, indicated in green). (3) After washing, bound antibody-antigen complexes were eluted, the eluate was desalted into ammonium bicarbonate and lyophilized. **d**, WB analysis of the DLB brain extract after ID with 2F12 or 46-4 and the corresponding IPs. Recombinant αSyn was loaded as a positive control, and proteins detected with C-20 (left). Identical blots were developed with rabbit IgG (right). **e**, WB analysis of the PiD brain extract after ID with Tau5 or 46-4, and the corresponding IPs. Recombinant tau (hT40) was loaded as a positive control, and proteins detected with K9JA (left). Identical blots were developed with rabbit IgG (right). **f-h**, iNs expressing (CR-C; circles) or lacking PrP (CR-*PRNP;* diamonds) were incubated without (Media; black) or with extracts from AD (**f**; blue), DLB (**g**; orange) or PiD (**h**; pink) extracts and neurite length measured using live-cell imaging. Data indicate the mean ± SEM of 4 images taken from 3 wells and are representative of three independent experiments. For **b**, **d** and **e**, molecular weight markers (in kDa) are indicated on the right.

Application	Code	Age	Gender	PMI (h)	CLDx	NPDx	Braak	Thal	ADNC A	ADNC B	ADNC C	LBD Braak	Tissue Region(s)
Biochemistry and Toxicity Studies	NDC	92	M	18	Normal	Normal	II	0	0	1	0	-	Superior/Inferior Frontal Cortex
	AD1	68	F	36	AD	AD	VI	-	-	-	-	-	Temporal Cortex
	AD2	66	F	28	FTD/AD	AD	VI	5	3	3	3	-	Superior/Inferior Temporal Cortex
	DLB1	79	F	24	PD	DLB	IV	3	2	2	2	6	Superior/Inferior Frontal Cortex
	DLB2	88	M	24	PD	DLB	III	0	0	2	0	5	Superior/Inferior Frontal Cortex
	PiD1	79	M	24	FTD	PiD	0	0	0	0	0	-	Temporal Pole
	PiD2	67	M	40	FTD	PiD	II	3	2	1	1	-	Temporal Pole
Array Tomography Studies	AD3	85	F	80	AD	AD	VI	5	-	-	-	-	Middle/Inferior Temporal Cortex
	AD4	89	F	96	AD	AD	VI	5	-	-	-	-	Middle/Inferior Temporal Cortex
	AD5	85	F	45	AD	AD	VI	-	-	-	-	-	Middle/Inferior Temporal Cortex
	DLB3	83	F	24	DLB	DLB	III	-	2	3	2	6	Middle/Inferior Temporal Cortex
	DLB4	77	M	24-48	DLB	DLB	III	-	1	2	1	5	Middle/Inferior Temporal Cortex
	DLB5	62	F	9	DLB	DLB	IV	-	2	2	2	5	Middle/Inferior Temporal Cortex
	FTD1	78	F	76	FTD	CBD	-	-	-	-	-	-	Middle/Inferior Temporal Cortex
	FTD2	76	M	46	FTD	PSP	-	1	-	-	-	-	Middle/Inferior Temporal Cortex
	FTD3	88	F	73	FTD	GGT	-	-	-	-	-	-	Middle/Inferior Temporal Cortex

Supplementary Table 1. Demographic details of the cases used in toxicity and array tomography studies. AD, Alzheimer's disease; ADNC, Alzheimer's disease neuropathologic changes; ADRC, Alzheimer's Disease Research Center; Braak, Braak staging of neurofibrillary tangles; CBD, corticobasal degeneration; CLDx, clinical diagnosis; DLB, dementia with Lewy body; F, female; FTD, frontotemporal dementia; GGT, globular glial tauopathy; LBD Braak, Braak staging of Lewy bodies; M, male; NPDx – neuropathology diagnosis; PD, Parkinson's disease; PiD, Pick's disease; PMI, postmortem interval; PSP, progressive supranuclear palsy; Thal, Thal amyloid phase; -, data not available or not collected.

Target	Antibody	Epitope	Clonality (Isotype)	Dilution (Assay)	Source	Reference
α-synuclein	2F12	125-135	Mouse mAb (IgG2)	100 µg/ml (IP); 2 µg/ml (ICC); 0.5 µg/ml (BA)	Bartels Lab	(Dettmer et al., 2013)
α-synuclein	4B12	103-108	Mouse mAb (IgG1)	2 µg/ml (OCW)	ThermoFisher	(EbrahimiFakhari et al., 2011)
α-synuclein	C-20	α-Syn C-terminus	Rabbit pAb (IgG)	1:750 (WB)	Santa Cruz	(Abeliovich et al., 2000)
α-synuclein	SOY1	91-100	Mouse mAb (IgG2)	7 µg/ml (ELISA)	Bartels Lab	(Dettmer et al., 2015)
α-synuclein	Syn211	121-125	Mouse mAb (IgG1κ	1:100 (AT)	ThermoFisher	(Giasson et al., 2000)
Amyloid-β	21F12	Aβ ter. at Ala42	Mouse mAb (IgG1)	1 µg/ml (MSD-IA)	Elan	(Johnson-Wood et al., 1997)
Amyloid-β	266	16-23	Mouse mAb (IgG1)	1 µg/ml (MSD-IA)	Elan	(Johnson-Wood et al., 1997)
Amyloid-β	3D6	1-5	Mouse mAb (IgG1)	2 µg/ml (ICC); 2 µg/ml (OCW); 0.5 µg/ml (BA)	Elan	(Johnson-Wood et al., 1997)
Amyloid-β	AW7	Pan-Aβ	Rabbit pAb (IgG)	1:50 (IP)	Walsh Lab	(McDonald et al., 2012)
Amyloid-β	6E10	3-8	Mouse mAb (IgG1)	AT (1:100)	BioLegend	(Wisniewski et al., 1998)
APP	C1/6.1	676-695	Mouse mAb (IgG1)	1 µg/ml (WB)	BioLegend	(Mathews et al., 2002)
β-III-Tubulin	D71G9	β-III-Tub. C-terminus	Rabbit mAb (IgG)	1:4000 (WB); 1:500 (OCW)	Cell Signaling	(Geisert et al., 1989)
GAPDH	D16H11	GAPDH C-terminus	Rabbit mAb (IgG)	1:4000 (WB)	Cell Signaling	(Zheng et al., 2003)
GFP	D5.1	GFP N-terminus	Rabbit mAb (IgG)	1:500 (ICC); 1:2000 (WB)	Cell Signaling	(Chiou et al., 2014)
HIV	46-4	HIV gp120	Mouse mAb (IgG1)	5 µg/ml (IP); 3 µg/ml (ABE)	ATCC	(Wang et al., 2007)
MAP2	AB15452	Full-length MAP2	Chicken pAb (IgY)	1:200 (ICC)	Millipore	(Corbett et al., 2015)
PrP	6D11	93-109	Mouse mAb (IgG2)	4 µg/ml (ICC)	BioLegend	(Pankiewicz et al., 2006)
PrP	D18	132-156	Human mAb (IgG1)	1 µg/ml (ICC); 0.5 µg/ml (WB)	Harris Lab	(Williamson et al., 1998)
PrP	ICSM18	143-153	Mouse mAb (IgG1)	1 µg/ml (WB); 2 µg/ml (OCW)	Collinge Lab	(Khalili-Shirazi et al., 1992)
PrP	ICSM35	93-105	Mouse mAb (IgG2)	3 µg/ml (ABE)	Collinge Lab	(Khalili-Shirazi et al., 1992)
PrP	MI-0131	23-51	Human mAb (IgG1)	3 µg/ml (ABE)	MedImmune	(Ondrejcak et al., 2018)
PrP	SAF32	58-89	Mouse mAb (IgG2)	1 µg/ml (WB)	Cayman	(Demart et al., 1999)
PrP	EP1802Y	200-300	Rabbit mAb (IgG)	1:50 (AT)	Abcam	(Groveman et al., 2017)
Synaptophysin	Sy38	C-terminus	Goat pAb (IgG)	1:20 (AT)	R & D	(Wang et al., 2017)
Tau	BT2	194-198	Mouse mAb (IgG1)	2.5 µg/ml (ELISA)	ThermoFisher	(Mercken et al., 1992)
Tau	HJ8.5	25-30	Mouse mAb (IgG2)	2 µg/ml (ICC); 2 µg/ml (OCW); 0.2 µg/ml (BA)	Holtzman Lab	(Yanamandra et al., 2013)
Tau	K9JA	243-441	Rabbit pAb (IgG)	1 µg/ml (WB)	Dako	(Wang et al., 2007)
Tau	Tau12	6-18	Mouse mAb (IgG1)	2.5 µg/ml (ELISA)	Millipore	(Ghoshal et al., 2002)
Tau	Tau5	210-241	Mouse mAb (IgG1)	5 µg/ml (IP); 2.5 µg/ml (ELISA)	BioLegend	(Carmel et al., 1996)
Tau (p)	AT8	pS202/205	Mouse mAb (IgG1)	1:50 (AT)	ThermoFisher	(Goedert et al., 1995)
N/A	011-000-003	Whole IgG	Rabbit pAb (IgG)	1 µg/ml (WB)	Jackson	N/A

Supplementary Table 2. Primary antibodies and their antigens, dilutions and sources. mAb, monoclonal antibody; pAb, polyclonal antibody; IP, immunoprecipitation; ICC, immunocytochemistry; BA, PrP binding assay; OCW, on-cell Western blot; WB, Western blot; ELISA, enzyme-linked immunosorbent assay; MSD-IA, MesoScale Discovery immunoassay; ABE, antibody blocking experiments; AT, array tomography.
